# Shedding‐weighted network approaches for understanding tuberculosis maintenance in multihost systems using camera traps

**DOI:** 10.1002/eap.70274

**Published:** 2026-06-04

**Authors:** Patricia Barroso, Matthew J. Silk, Alberto Perelló, Ana Balseiro, David Relimpio, Nuno Santos, Christian Gortázar

**Affiliations:** ^1^ Departamento de Sanidad Animal, Facultad de Veterinaria Universidad de León León Spain; ^2^ Institute of Ecology and Evolution, School of Biological Sciences, University of Edinburgh Edinburgh UK; ^3^ SaBio, Health and Biotechnology Group, Instituto de Investigación en Recursos Cinegéticos (IREC) CSIC‐UCLM‐JCCM Ciudad Real Spain; ^4^ Department of Animal Health Mountain Livestock Institute (IGM‐CSIC) León Spain; ^5^ CIBIO, Centro de Investigação em Biodiversidade e Recursos Genéticos, InBIO Laboratório Associado, Universidade do Porto Vairão Portugal; ^6^ BIOPOLIS Program in Genomics, Biodiversity and Land Planning, CIBIO Vairão Portugal

**Keywords:** ecological networks, local centrality, *Mycobacterium tuberculosis* complex, shared diseases, shedding, wildlife‐livestock interface

## Abstract

Understanding the dynamics of multihost pathogens, such as *Mycobacterium tuberculosis* complex (MTC), requires considering not only host interaction patterns but also variation in infectiousness across species. Network analysis is a useful tool to assess contact structure and disease risk, but it often depends on invasive methods. Camera trapping offers a noninvasive alternative to build co‐occurrence networks in complex communities. In this study, we applied a novel approach that integrates shedding into ecological networks, weighing links between species pairs according to their co‐occurrence frequency and shedding capacity, to evaluate tuberculosis (TB) risk across 18 study sites in the Iberian Peninsula. At the community level, TB risk was positively associated with mean strength‐out, a local centrality measure of the frequency of interspecific contacts and their infectious potential. Species‐specific models revealed that community TB risk increased with the strength‐out of wild boar, red deer, and cattle, and with the closeness of red fox and badger. Furthermore, the community TB risk was jointly explained by shedding‐weighted connectivity and spatial aggregation of wild boar, whereas red deer mainly contributed through their local abundance. In contrast, shedding‐weighted centrality of badgers, foxes, and cattle explained TB risk, suggesting that ecological and management factors may influence TB spread. We defined epidemiological scenarios according to latitude, population factors, and infection pressure. These findings highlight the importance of including host infectiousness in ecological network analyses, as well as combining centrality measures and population data to understand and manage TB risk in complex host communities, with potential applications to other wildlife diseases and multihost systems.

## INTRODUCTION

Many animal pathogens have a broad host range and circulate within complex ecological communities, where multiple wild and domestic species, and even humans, interact and serve as hosts, thereby facilitating pathogen transmission (VerCauteren et al., 2021). In these contexts, the coexistence of multiple competent and noncompetent hosts can modify disease dynamics, leading to outcomes such as amplification or dilution effects (Rohr et al., 2019). However, infectious disease dynamics are influenced by a complex interplay of factors (McCallum et al., 2017). Transmission depends first on exposure of susceptible hosts, primarily influenced by the spatiotemporal overlap among species. This overlap is itself determined by host behavioral and ecological traits (e.g., social behavior, feeding, habitat preferences) as well as environmental factors like resource distribution and habitat characteristics (Tompkins et al., 2011). Second, transmission depends on host infectiousness, determined by their shedding patterns. Among these factors, pathogen shedding is particularly relevant, as it determines a host's ability to serve as a source of infection. In indirectly transmitted diseases like animal tuberculosis (TB), transmission occurs through the ingestion or inhalation of pathogens released into the environment by infected hosts. In contrast, for directly transmitted diseases, shedding facilitates spread through close contact (Barasona, Torres, et al., 2017; Clarke et al., 2022; King et al., 2015).

TB is a multihost disease caused by *Mycobacterium bovis* and other members of the *Mycobacterium tuberculosis* complex (MTC), with significant economic and public health implications (Fitzgerald & Kaneene, 2013). Transmission occurs both directly and indirectly through contaminated shared resources such as water or food (Ferreira et al., 2023; Fitzgerald & Kaneene, 2013; Kukielka et al., 2013). Environmental contamination results from MTC shedding via nasal, oral, fecal, or urinary routes, which varies in frequency and intensity among host species (Barasona, Torres, et al., 2017). The Eurasian wild boar (*Sus scrofa*) and red deer (*Cervus elaphus*) are primary wildlife hosts and efficient shedders, especially when presenting clinical disease, maintaining and transmitting MTC even in the absence of other potential hosts (Gortázar et al., 2012; Santos et al., 2012). The European badger (*Meles meles*), a recognized maintenance host in the United Kingdom and Ireland, may contribute to TB persistence in some regions, although its shedding is intermittent and variable (Acevedo et al., 2019; Blanco Vázquez et al., 2021; Gavier‐Widen et al., 2001; Muñoz‐Mendoza et al., 2013). Other wild ungulates, such as the roe deer (*Capreolus capreolus*) and fallow deer (*Dama dama*), show lower prevalence and shedding evidence (Balseiro et al., 2009; Barroso et al., 2020). The red fox (*Vulpes vulpes*) and the Egyptian mongoose (*Herpestes ichneumon*) have been sporadically found infected, but their epidemiological roles remain poorly understood (Ferreras‐Colino et al., 2023; Martín‐Atance et al., 2005; Millán et al., 2008; Muñoz‐Mendoza et al., 2013). Data on shedding in other species like mouflon (*Ovis aries musimon*) are scarce, but infection has been reported (García‐Bocanegra et al., 2012). Among livestock, cattle are a major reservoir of TB, while goats are also recognized as a relevant species and included in eradication programs in areas of contact with cattle (Gortazar et al., 2011; MAPA, 2017; Napp et al., 2013). Both species can shed bacteria via multiple routes, even subclinically, while pigs and sheep have lower shedding potential (Cano‐Terriza et al., 2018; Di Marco et al., 2012; Munoz‐Mendoza et al., 2016; Napp et al., 2013).

Network analysis is a useful approach to understand disease spread in multihost systems, as it facilitates the identification of individuals or species involved in pathogen transmission, potential hotspots and associated risk factors (Luis et al., 2015; Martínez‐López et al., 2009; Silk et al., 2017). Traditionally, such analyses rely on direct observation or biologging technologies that may involve some disturbance to wild animals (Triguero‐Ocaña et al., 2020; White et al., 2017). In contrast, camera trapping offers a noninvasive alternative for wildlife surveillance with lower impact, enabling the construction of social networks when individuals can be identified, or ecological networks based on species co‐occurrence (Luis et al., 2015; Vázquez et al., 2007). While most network‐based epidemiological studies focus primarily on describing interaction structure, their combination with host infectiousness or pathogen shedding remains unexplored. The contribution of each host species to disease maintenance is closely linked to its shedding potential, which ultimately determines its capacity to contaminate the environment and transmit infection (Barasona, Vicente, et al., 2017; Risco et al., 2019; Santos et al., 2015). Accounting for variation in pathogen shedding across species in network analyses could improve the forecasting of disease dynamics and help to better identify high‐risk species or taxonomic groups.

In this context, we applied network analysis to camera trap data from 18 sites across the Iberian Peninsula, collected as part of a pilot monitoring project for integrated wildlife surveillance. The nodes in the networks represent species rather than individuals, so edges correspond to spatiotemporal co‐occurrence between species at camera stations, serving as a proxy for potential indirect contact, as in previous co‐occurrence‐based network studies (Barroso et al., 2023; Barroso & Gortázar, 2024). Network measures were weighted by species‐specific shedding potential derived from field data and estimations, thereby extending earlier approaches by incorporating heterogeneity in infectiousness in a novel way to better address potential MTC transmission dynamics. We aimed to (1) identify which species shedding‐weighted centrality measures best explain TB risk, (2) identify species with a more significant role in TB transmission (i.e., higher infection pressure), (3) assess the relative contribution of both shedding‐weighted centrality and population factors to TB risk, and (4) identify high‐risk study sites.

## METHODS

### Study area

**FIGURE 1:**
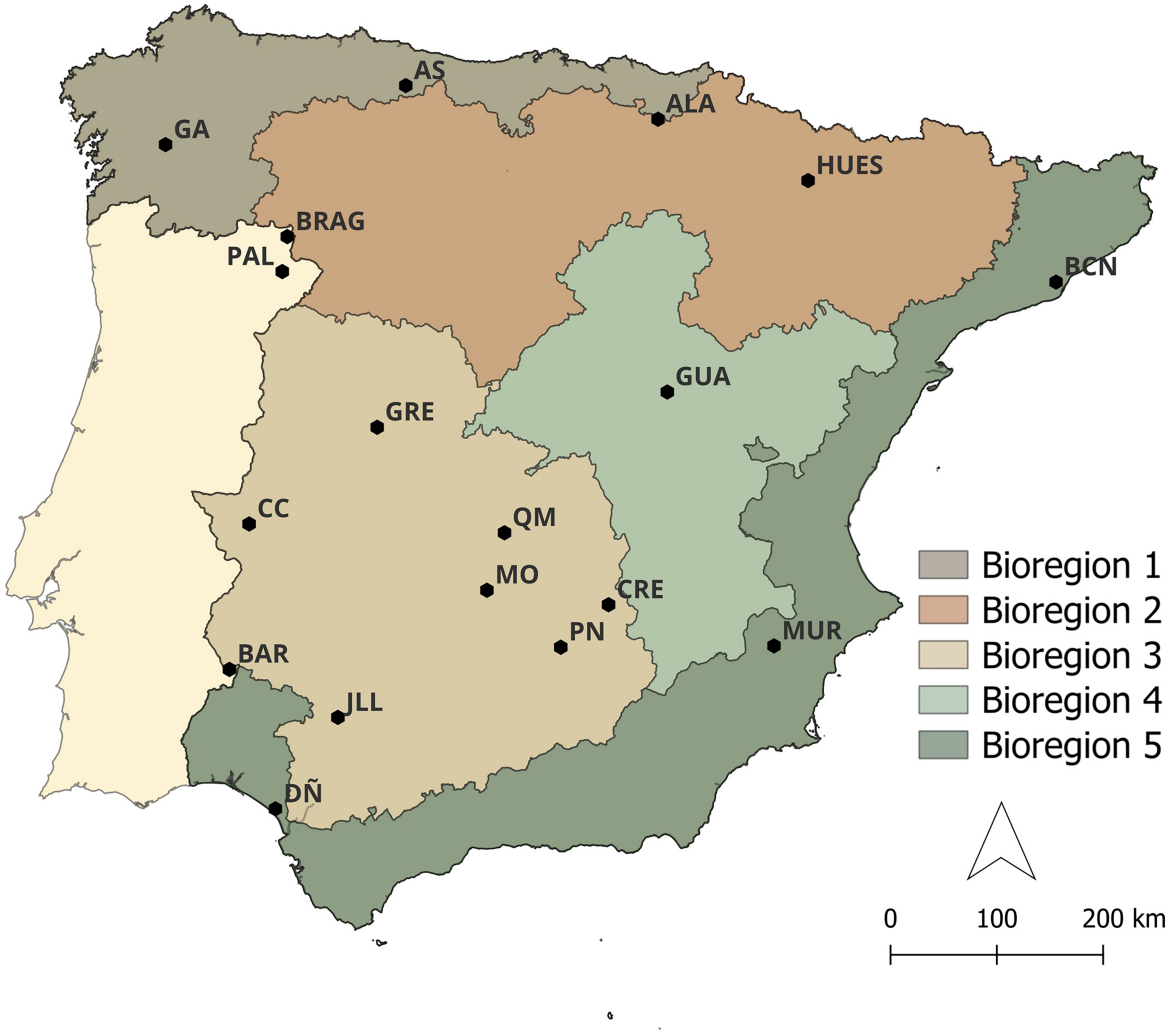
Map of the 18 study sites with a division of the Iberian Peninsula into five large bioregions (1–5) according to the Spanish Wildlife Disease Surveillance Scheme. The most epidemiologically relevant host present at each site is displayed.

Eighteen study sites were selected as they encompassed the main habitats, climates, and wildlife management systems of the Iberian Peninsula, ensuring a representative distribution across its main bioclimatic units (Figure 1) (Barroso et al., 2023; MAPA, 2025).

### Sample collection and enzyme‐linked immunosorbent assay (ELISA)

The Eurasian wild boar was considered the indicator species for the TB status of each host community. In multihost systems, abundant and highly exposed hosts are often monitored as indicators of community‐wide pathogen circulation when comprehensive multispecies surveillance is logistically unfeasible (Ferrara & Tejeda, 2024; Ojeyinka & Omaghomi, 2024). MTC provides several examples of this approach, including the use of badgers in the United Kingdom and brushtail possums in New Zealand, as well as polar bears in Arctic ecosystems, to infer broader transmission dynamics (Anderson et al., 2013; Moustakas & Evans, 2015; Tschritter et al., 2023). In Europe, the wild boar is one of the most widespread and frequently infected wildlife hosts, with a wide distribution, high abundance, and extensive use of shared resources (Barasona et al., 2014; Naranjo et al., 2008; Torres et al., 2020). Consequently, TB infection or exposure in wild boar can inform community‐level infection pressure rather than directly estimate species‐specific prevalence. Recent studies have shown that diagnostic testing in wild boar provides reasonably accurate estimates of true TB prevalence and captures spatial variation across multihost systems, supporting their use in ecological and surveillance analyses (Cardoso et al., 2024; Reis et al., 2021). From a practical perspective, wild boar mounts strong and detectable antibody responses to MTC, facilitating reliable serological surveillance and consistent comparison among sites (Aurtenetxe et al., 2008; Varela‐Castro et al., 2020).

A total of 1109 blood samples were obtained from the endocranial venous sinus of hunter‐harvested wild boar at our 18 study sites between 2020 and 2024. Sera were obtained by centrifugation at 400*g* for 5 min and tested for antibodies against MTC by using an in‐house indirect P22‐ELISA, as previously described by Thomas et al. (2019). Positive and negative controls were included in each plate and were obtained from TB‐confirmed and TB‐free animals, respectively.

### Camera trapping and weighted directed network analysis

A grid of approximately 20 unbaited camera traps (range: 14–24; Browning Strike Force HD ProX, Browning Arms Company, Morgan, Utah, USA) was deployed at each study site for 40 days in 2022 (see Appendix S1: Section S1). Cameras were placed 50 cm above ground, facing north, and operated 24 h daily, capturing eight consecutive images per activation, with a one‐second delay between activations. Independent captures were defined as pictures of individuals taken more than 10 min apart (Barroso et al., 2023). Cameras operativing for less than 5 days were excluded from the analysis, and local effort was standardised to 450 camera‐days. Data were filtered to include species most relevant to TB epidemiology in the Iberian Peninsula, namely wild boar, red deer, fallow deer, roe deer, European badger, mouflon, red fox, Egyptian mongoose, small ruminants (namely sheep and goats), and cattle (MAPA, 2017, 2025).

Spatiotemporal coincidences between species were recorded when individuals were photo‐captured in the same camera within 24 h, according to the estimated MTC environmental persistence (Kukielka et al., 2013; Niedballa et al., 2019). For each site, we built a directed and weighted static contact network, with species as nodes and spatiotemporal coincidences between species pairs as weighted links. Although co‐occurrence is bidirectional by definition (i.e., if species A co‐occurs with B, the reverse is equally true), the direction and weight of each link depended on the shedding (infectiousness) of the source species and the frequency of co‐occurrence with the recipient species (i.e., the potential transmission risk from species A to B differs from B to A). Therefore, our final networks were weighted and directed, with edge weights corresponding to a product of the shedding of one species (source node) and the number of co‐occurrences with its paired species (recipient node). Infectiousness values were obtained from excretion rates (CFU/mL) reported in an ongoing research by our group (Jiménez‐Ruiz et al. 2026). For most species lacking shedding data (i.e., Egyptian mongoose, roe deer, mouflon, and red fox), we assigned an approximate value equal to one‐tenth of the lowest empirically observed shedding rate (i.e., European badger, 1.26 CFU/mL). This choice avoids overestimating their contribution while allowing their inclusion in the analysis, ensuring their contribution is accounted for despite missing data (see Table 1 for detailed rationale behind the assignment of approximate values). For small ruminants, given their potential epidemiological similarity to cattle, we assigned a shedding value corresponding to approximately 60% of the cattle estimate (0.81 vs. 1.38) as a conservative proxy. Table 1 displays the shedding values used in the network analyses, including empirically derived data and the rationale behind the assignment of approximate values for species lacking direct measurements. While these conservative estimates could be refined in future studies, overall, sensitivity analyses (Appendix S1: Section S2) confirmed that our main conclusions were robust to variation in these assigned values. Egyptian mongoose and mouflon were included in the visual network graphs; however, due to their limited occurrence (in only three and two sites, respectively), they were excluded from subsequent analyses to ensure robustness and consistency of the results.

**TABLE 1:** *Mycobacterium tuberculosis* complex (MTC) shedding values by species and reference information.

Species	MTC concentration (CFU/mL)	Reference	Reason
Cattle	1.385	Jiménez‐Ruiz et al. (2026)	Empirical data
Wild boar	2.872	Jiménez‐Ruiz et al. (2026)	Empirical data
Red deer	2.038	Jiménez‐Ruiz et al. (2026)	Empirical data
Fallow deer	1.494	Jiménez‐Ruiz et al. (2026)	Empirical data
European badger	1.265	Jiménez‐Ruiz et al. (2026)	Empirical data
Roe deer	0.13	Extrapolated data (Balseiro et al., 2009; García‐Bocanegra et al., 2012)	Informed by known prevalence, reported generalization capacity, and the presence of only sporadic or isolated cases, suggesting limited transmission potential. Consequently, it was assigned a minimum value. The minimum value was equal to one‐tenth of the lowest empirically observed shedding rate (European badger, 1.26 CFU/mL).
Red fox	0.13	Extrapolated data (Martín‐Atance et al., 2005; Millán et al., 2008)	Informed by known prevalence, reported generalization capacity, and the presence of only sporadic or isolated cases, suggesting limited transmission potential. Consequently, it was assigned a minimum value. The minimum value was equal to one‐tenth of the lowest empirically observed shedding rate (European badger, 1.26 CFU/mL).
Egyptian mongoose	0.13	Extrapolated data (Ferreras‐Colino et al., 2023)	Informed by known prevalence, reported generalization capacity, and the presence of only sporadic or isolated cases, suggesting limited transmission potential. Consequently, it was assigned a minimum value. The minimum value was equal to one‐tenth of the lowest empirically observed shedding rate (European badger, 1.26 CFU/mL).
Mouflon	0.13	Extrapolated data (García‐Bocanegra et al., 2012)	Informed by known prevalence, reported generalization capacity, and the presence of only sporadic or isolated cases, suggesting limited transmission potential. Consequently, it was assigned a minimum value. The minimum value was equal to one‐tenth of the lowest empirically observed shedding rate (European badger, 1.26 CFU/mL).
Domestic small ruminants (sheep and goat)	0.81	Extrapolated data (Jiménez‐Martín et al., 2024; Muñoz Mendoza et al., 2012)	Despite their lower overall prevalence, they shed MTC via multiple routes and may maintain infection in mixed‐species systems. Therefore, they were assigned an intermediate value, closer to that of cattle (~60% of the cattle estimate).

Abbreviation: CFU, colony‐forming units.

**FIGURE 2:**
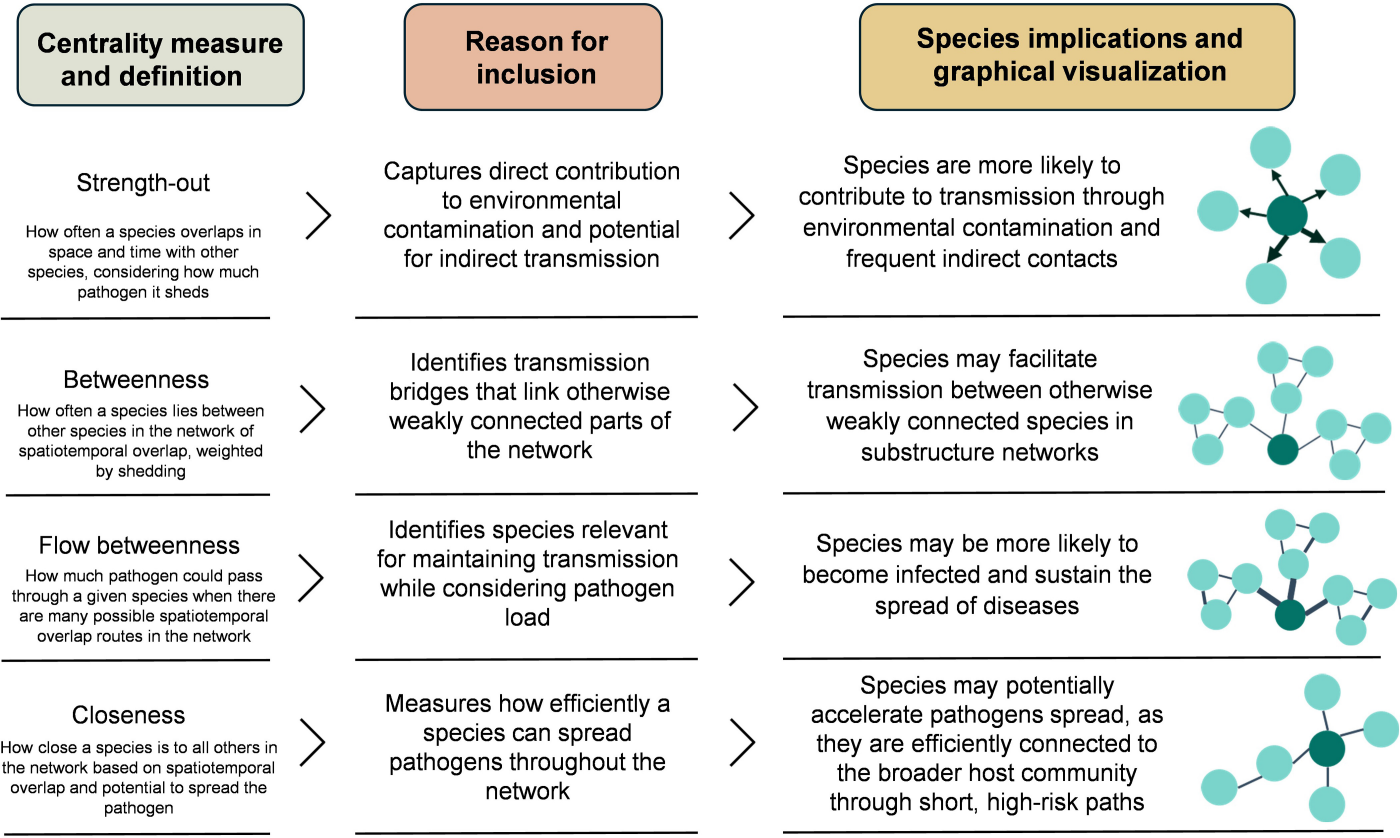
Centrality measures calculated from ecological networks including their definitions, rationale for inclusion in the analyses, and implications for pathogen transmission. Each measure is illustrated graphically with the most central node (species) highlighted in a darker color.

We calculated four shedding‐weighted centrality measures (strength‐out, closeness, betweenness, and flow betweenness) using the *igraph* and *sna* R packages. A summary of these measures, their definitions, rationale for inclusion in the analyses, and their implications for disease transmission is provided in Figure 2. By including these four measures, we aimed to capture both the local contribution of each species to transmission, through its direct contacts and shedding (e.g., strength‐out and closeness), and its broader role across the network (e.g., betweenness and flow betweenness).

### FBII and the aggregation index of wild boar and wild ruminants

Wildlife signs were recorded along randomly selected transects, with a total of 1000 m per study site. Each site included five linear transects, each measuring 200 m in length and 1 m in width. These transects were further divided into 10‐m sectors. Sign frequency, used for abundance estimation, was defined as the average number of 10‐m sectors with target species signs (e.g., wild boar droppings, tracks, and rootings, or European badger latrines). These frequencies were then used to calculate the frequency‐based indirect index of abundance (FBII) for wild boar, wild carnivores, and wild ruminants (Acevedo et al., 2007; Vicente et al., 2004). In addition, to calculate the spatial aggregation index of wild boar, we transformed the sign frequency data according to the runs test statistic. The absolute value of *Z* was used as the index of sign aggregation, with higher values indicating stronger clustering (Acevedo et al., 2007). The wild boar aggregation index is a proxy of their spatial behavior, which may affect TB transmission risk (Vicente et al., 2004).

### Statistical analysis

#### Models

We fitted a set of generalized linear mixed models (GLMMs) for each of the first three aims. The models were run at the individual level (*n* = 1109), that is, individual seropositivity to TB of the wild boar tested.

For the first aim (i.e., to identify the shedding‐weighted species centrality measures that best explain TB risk), GLMMs were fitted to assess the relationship between community TB risk and four community‐level weighted centrality measures: mean flow betweenness, mean betweenness, mean closeness, and mean strength‐out. Due to collinearity among these centrality measures (variance inflation factor > 3), each measure was modeled separately (i.e., four distinct models). In all models, the response variable was individual TB seropositivity in wild boar (binomial: positive/negative; used as a proxy to estimate the TB risk of the whole host community), the fixed effect was the respective mean centrality measure, and the random effect was the study site. Although the centrality measures were calculated at the site level (i.e., identical for all individuals within a site), the inclusion of the study site as a random intercept accounts for nonindependence of individual wild boar observations within sites and captures unmeasured site‐specific heterogeneity influencing TB seropositivity that is not explained by site‐level fixed effects included in the models.

For the second aim (i.e., to assess whether certain species play a more significant role in TB transmission), we tested the relationship between TB risk and each species‐level weighted centrality measure. For this, models were fitted separately for each species and each centrality measure. In all models, the response variable was individual TB seropositivity in wild boar (used as a proxy to estimate the TB risk of the whole host community), the fixed effect was the corresponding centrality measure of each species (flow betweenness, betweenness, closeness, and strength‐out), and the random effect was the study site.

Regarding the third aim, to assess the relative contribution of population‐related factors and shedding‐weighted species centrality to community TB risk, we fitted a set of GLMMs for each species previously identified as relevant to TB transmission (i.e., those displaying a significant relationship between centrality measures and TB seropositivity in the prior analysis). In these models, the response variable was individual TB seropositivity in wild boar (used as a proxy to estimate the TB risk of the whole host community). Fixed effects included a population‐related factor (either aggregation index or relative abundance) and the weighted centrality measure that showed the strongest predictive power for that species in the previous step. The study site was included as a random effect. Models were fitted separately for each species. This approach allowed us to assess whether TB risk was better explained by population factors, network position, or a combination of both. For wild boar, strong correlations were found between centrality measures, FBII, and trapping rates (Spearman's *R* = 0.80, *p* < 0.01; *R* = 0.73, *p* < 0.01), so we used the aggregation index instead in the models. For red deer, high correlation between centrality measures and trapping rates (Spearman's *R* = 0.93, *p* < 0.01) led us to include FBII in the models. For wild carnivores and cattle, no collinearity was detected, so trapping rate was used as a species‐specific measure of relative abundance (O'Brien, 2011).

Model selection was based on corrected Akaike's information criterion (AIC_c_). All the models were fitted using the *lme4* package in R software 4.0.2. For each model, marginal *R* ^2^ (variance explained by fixed effects) and conditional *R* ^2^ (variance explained by both fixed and random effects) were calculated. Statistical significance was set at *p* ≤ 0.050 or adjusted according to Bonferroni corrections for multiple comparisons (*p* ≤ 0.013) when applicable. Statistical inference was based on the Wald *z*‐test for fixed effects. Model fit was assessed through residual diagnostics, and conditional *R* ^2^ values were used to evaluate model explanatory power.

#### Principal component analysis

To characterize study sites in terms of species connectivity, species abundance, and TB seroprevalence, we performed a principal component analysis (PCA). This analysis aimed at grouping sites based on shared features that may underlie elevated TB risk. The variables included were wild boar aggregation index, relative abundance of red deer, strength‐out for both wild boar, and red deer, TB seroprevalence at the study site level, and latitude. These variables were selected based on their relevance to TB risk, as identified in previous modeling steps. Data on other species were not included as they were not present in all study sites. Latitude was included to account for known spatial gradients in TB distribution across the Iberian Peninsula (Barroso et al., 2023; Vicente et al., 2006). The resulting factors with eigenvalues >1 were selected. Individual scores on the first two principal components were extracted and used to perform a *k*‐means clustering analysis. Finally, the mean values of the original variables within each cluster were calculated to characterize the identified groups. The faxtra library in R software was used.

## RESULTS

**FIGURE 3:**
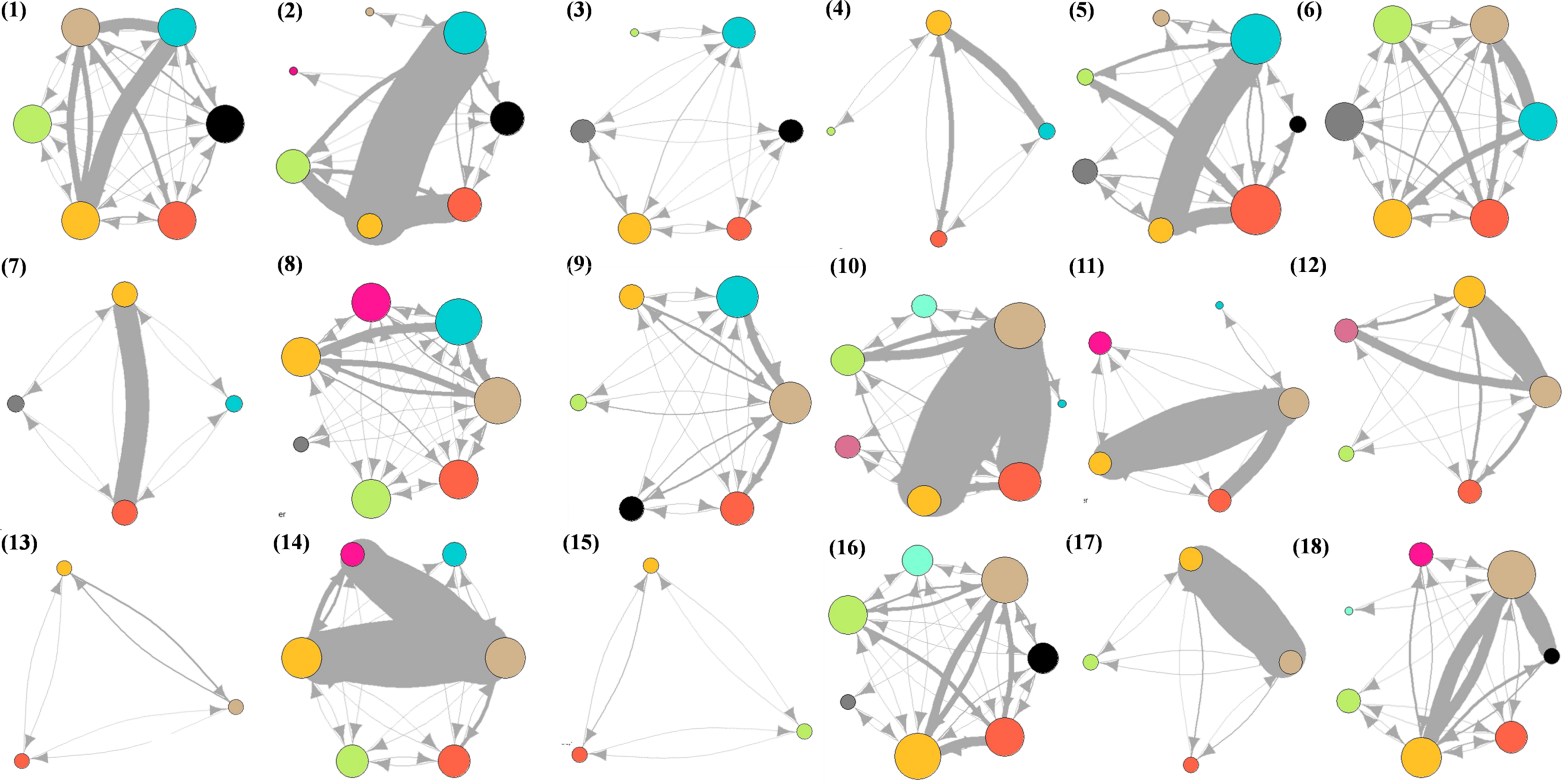
Ecological networks of the spatiotemporal coincides among the most epidemiologically relevant hosts for the *Mycobacterium tuberculosis* complex in the Iberian Peninsula (red deer, fallow deer, roe deer, mouflon, wild boar, European badger, red fox, Egyptian mongoose, cattle, sheep, and goat), obtained by camera trap records in 18 sites in the Iberian Peninsula. Nodes represent different species and edges represent spatiotemporal coincidences recorded by camera traps between species pairs. The size of the nodes represents their degree, and the weight of the edges represents potential transmission risk, calculated as the product of the infectiousness of one species and the number of recorded spatiotemporal co‐occurrences with its paired species. Site codes (with edge weights standardized by the indicated divisor): 1. AS (Cantabrian Mountains; weight/5), 2. ALA (Llanada alavesa Valley; weight/5), 3. GA (Northern coast; weight/5), 4. HUES (Pre‐Pyrenees; weight/5), 5. PAL (Trás‐os‐Montes Region; weight/5), 6. BRAG (Trás‐os‐Montes Region; weight/5), 7. BCN (Catalonia coast; weight/5), 8. GUA (Iberian System; weight/5), 9. GRE (Central System; weight/5), 10. CC (San Pedro Mountains; weight/5), 11. QM (Toledo Mountains; weight/5), 12. MO (Guadiana Valley; weight/25), 13. CRE (Campo de Montiel Region; weight/5), 14. PN (Sierra Morena Mountains; weight/25), 15. MUR (Cordillera Bética Mountains; weight/5), 16. BAR (Baixo Alentejo Region; weight/5), 17. JLL (Northern Seville Mountains; weight/25), 18. DÑ (Guadalquivir Valley; weight/5).

Figure 3 displays the co‐occurrence networks for each study site for all the epidemiologically relevant species implicated in the transmission and maintenance of the MTC in the Iberian Peninsula. Overall, the red deer and wild boar exhibited the highest shedding‐weighted centrality across multiple study sites, with elevated strength‐out (frequency of spatiotemporal overlap of a focal species with others weighted by shedding), closeness (overall proximity of a focal species to other species in the network weighted by shedding), and flow betweenness (a measure of “flow” such as pathogen spread through a focal node) values (Appendix S1: Section S3). Cattle, roe deer, and red fox also displayed high shedding‐weighted connectivity in specific areas. Wild boar and cattle showed the highest betweenness (presence of a focal species on the shortest “path” connecting two other species weighted). Finally, the European badger had a higher flow betweenness in some study sites.

### Mean node centrality measures at the community level related to TB risk

The mean shedding‐weighted species centrality measures and TB seroprevalences by study site are shown in Table 2. The TB risk was significantly related to the mean strength‐out of the species (Log‐odds [LO]: 1.40; 95% CI: 0.33–2.48; *p*: 0.010). In contrast, closeness, betweenness, and flow betweenness were not statistically significant (Appendix S1: Section S4, Tables S19–S22). The model including mean strength‐out as the explanatory variable was identified as the best model (AIC_c_ 657.4, ΔAIC< 2; Appendix S1: Section S5, Table S23), and this variable explained the highest variance in the model (marginal *R* ^2^: 0.20). In summary, only overall mean strength‐out had a significant association with TB risk in the host community, with the consequent greater relevance of shedding‐weighted connections between species in the network (close spatiotemporal overlap rather than strictly direct interactions between species).

**TABLE 2:** Mean local and global shedding‐weighted species centrality measures, their coefficient of variation, and seroprevalence of tuberculosis (TB) for each of the 18 study sites included in this study.

Study site	Strength‐out		Closeness		Betweenness		Flow betweenness		TB seroprevalence
	Mean	VC	Mean	VC	Mean	VC	Mean	VC	
ALA	179.6	1.99	0.13	0.43	7.43	1.04	111.94	0.96	0
AS	110.58	1.16	1.95	0.64	4.00	1.26	128.35	0.66	9.33
BAR	75.31	1.14	1.20	0.78	4.43	1.45	130.66	0.87	0
BCN	62.03	1.76	1.00	0.91	1.75	1.35	5.32	1.29	0
BRAG	89.03	0.96	2.08	0.68	2.17	1.64	131.46	0.4	0
CC	254.24	1.59	1.86	0.93	4.14	2.65	153.47	1.94	68.75
CRE	14.81	0.89	1.58	0.84	0.33	1.73	1.80	0.79	0
DN	94.63	1.27	0.74	0.73	5.71	1.81	90.88	1.53	24.17
GA	10.25	1.6	0.08	0.35	4.50	1.35	10.82	0.94	2.67
GRE	43.99	1.54	1.98	0.77	3.33	2.45	54.19	1.65	12.20
GUA	61.36	1.3	1.00	0.81	4.57	1.68	86.57	0.97	0.91
HUES	42.64	1.84	1.88	1.09	1.50	2	5.23	1.73	0
JLL	1069.49	1.17	11.94	0.9	1.75	1.35	64.84	1.06	36
MO	623.87	1.33	2.54	0.56	2.40	2.24	240.36	1.37	18.31
MUR	10.05	1.15	2.45	1.08	0.67	1.73	2.67	0.67	0
PAL	110.2	1.57	0.46	0.27	5.57	1.44	98.75	1.06	0
PN	1403.76	1.21	12.43	0.87	3.33	2.45	913.94	1.84	47.46
QM	182.81	1.33	1.67	0.79	2.40	2.24	53.30	1.81	38.46

Abbreviation: VC, variation coefficient.

### Species relevant for TB transmission

**FIGURE 4:**
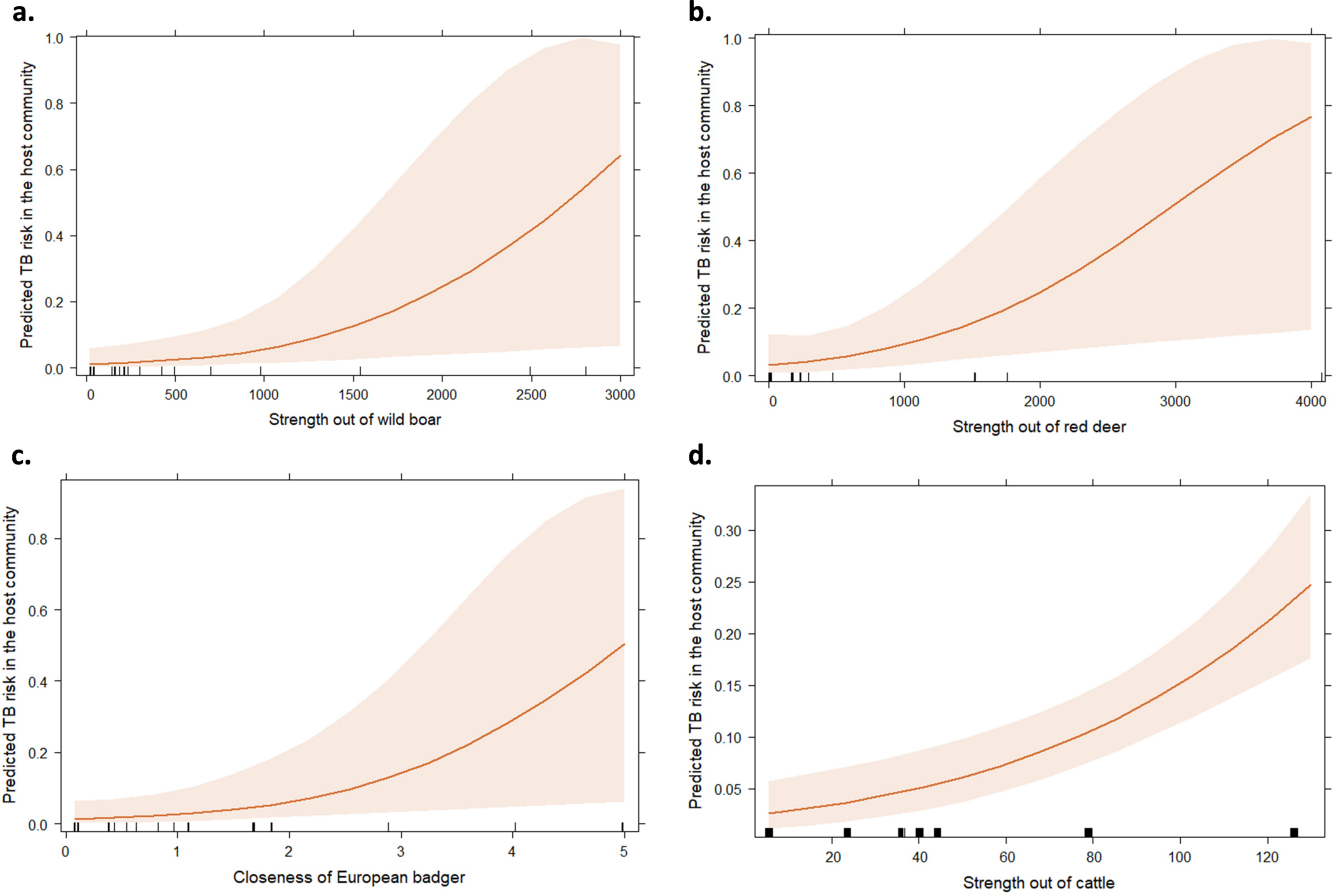
Predicted probability of tuberculosis (TB) depending on local centrality measures in the ecological networks: (a) the strength‐out of wild boar, (b) the strength‐out of red deer, (c) the closeness of European badgers, and (d) the strength‐out of cattle. Shaded bands represent the 95% CI.

When analyzing the importance of each species separately, strength‐out of wild boar showed a significant positive association with predicted community TB risk (LO: 1.35; 95% CI: 0.27–2.43; *p*: 0.010; Figure 4a; Appendix S1: Section S6, Table S34), being the best model (AIC_c_ = 658; ΔAIC < 2; Appendix S1: Section S5, Table S24). For red deer, their strength‐out significantly associated with TB risk (LO: 1.25; 95% CI: 0.23–2.27; *p*: 0.011; Figure 4b; Appendix S1: Section S6, Table S35). It was again identified as the best predictor (AIC = 629.2; Appendix S1: Section S5, Table S25), and although other centrality measures also displayed some potential, their associations with TB were weaker, leading us to select strength‐out as the most reliable predictor. Regarding red fox and badger, closeness showed a statistically significant relationship with TB risk (Figure 4c; Appendix S1: Section S6, Tables S36 and S37; red fox: LO: 1.54; 95% CI: 0.54–2.53; *p*: 0.002; badger: LO: 1.22; 95% CI: 0.21–2.23; *p*: 0.010), being the best model (Appendix S1: Section S5, Tables S26 and S27). In all these models, the study site accounted for a high proportion of variance (Appendix S1: Sections S6 and S7).

Regarding livestock, the strength‐out of cattle was positively related to TB risk (LO: 0.95; 95% CI: 0.56–1.34; *p* < 0.001; Figure 4d; Appendix S1: Section S6, Table S38).

The shedding‐weighted centrality measures of the other species tested were not statistically significant.

### The effects of species shedding‐weighted connectivity and population‐related factors on TB risk

For wild boar, a full model combined the most significant centrality measure for this species (strength‐out; see above) with wild boar aggregation. The best model (Appendix S1: Section S5, Table S29) revealed that the predicted TB risk in wild boar increased with both higher aggregation levels (LO: 1.34; 95% CI: 0.15–2.52; *p*: 0.021) and higher strength‐out (LO: 1.14; 95% CI: 0.17–2.11; *p*: 0.027; Appendix S1: Section S8, Table S39), indicating they had independent effects on community‐level TB risk.

In the case of red deer, the full model included strength‐out and the FBII relative abundance index. In the best model (also the full model; Appendix S1: Section S5, Table S30), the effect of FBII remained statistically significant (Appendix S1: Section S8, Table S40; LO: 0.94; 95% CI: 0.09–1.78; *p*: 0.029), while the effect of strength‐out was marginally significant (LO: 0.94; 95% CI: 0.09–1.78; *p*: 0.061). It indicates that at least part of the strength‐out effect was explained by abundance.

For wild carnivores and after model selection (Appendix S1: Section S5, Tables S31 and S32), TB risk was positively related to closeness centrality of both species (Appendix S1: Section S8, Tables S41 and S42; red fox: LO: 1.54; 95% CI: 0.54–2.53; *p*: 0.002; badger: LO: 1.21; 95% CI: 0.23–2.20; *p*: 0.016), while their trapping rates were either not retained in the best model (for red fox) or not significantly associated with TB risk (for badger; LO: −2.12; 95% CI: −4.74 to 0.50; *p*: 0.112). Similarly, in the case of cattle, model selection (Appendix S1: Section S5, Table S33) revealed that TB risk was positively associated with strength‐out (Appendix S1: Section S8, Table S43; LO: 0.95; 95% CI: 0.56–1.34; *p* < 0.001), whereas trapping rate was not included in the best model (ΔAIC = 0.74 but not statistically significant).

### PCA results

**FIGURE 5:**
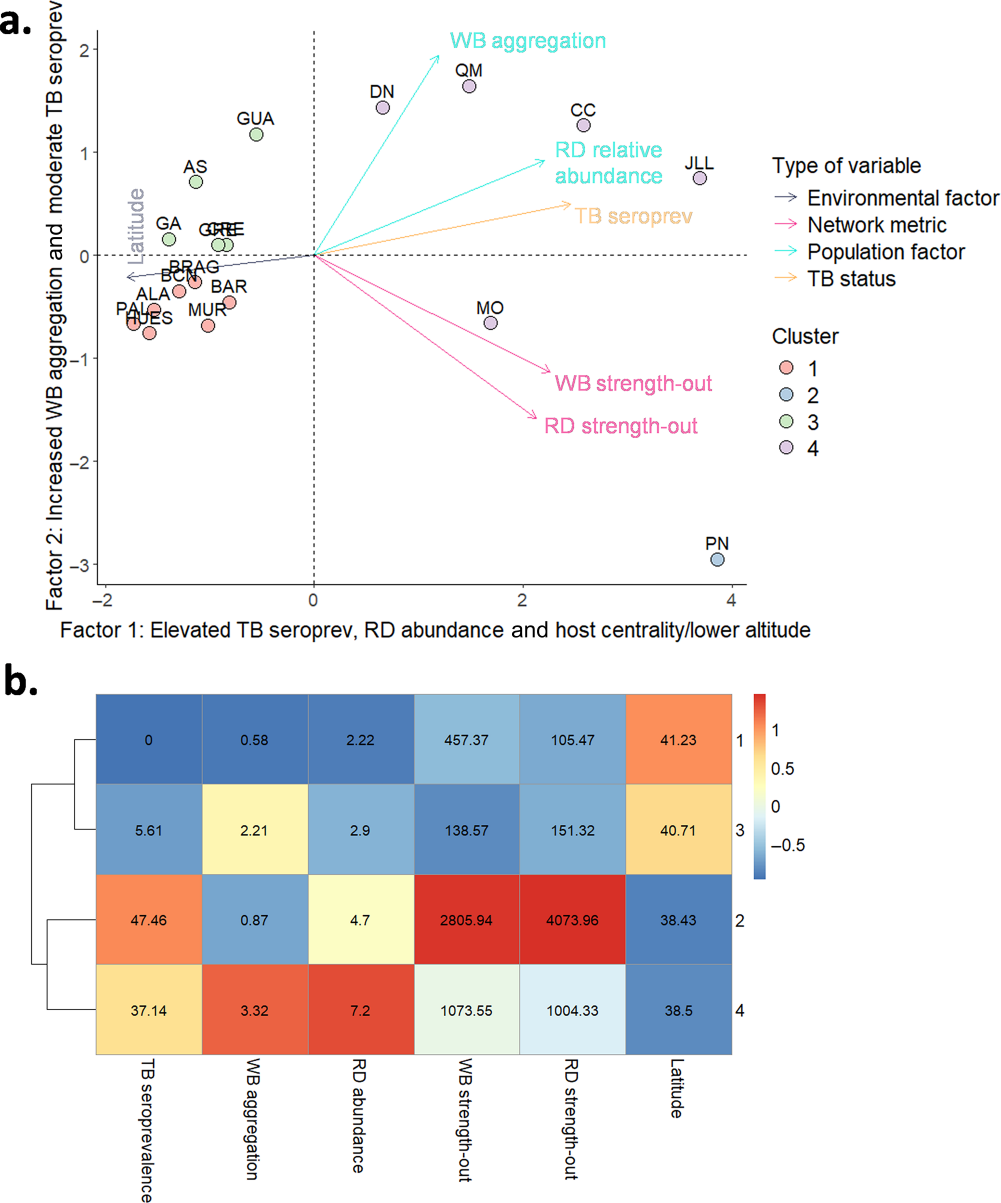
(a) Projections of the 18 study sites on the two‐dimensional space. The space was defined by principal component axes 1 and 2 (Factors 1 and 2) according to the study sites features in terms of location (latitude), host (red deer [RD] and wild boar [WB]) connectivity, RD abundance, WB aggregation, and tuberculosis (TB) seroprevalence. Arrows reflect the direction and contribution to the axes of each of the six parameters included in the principal component analysis (PCA). The four different clusters identified are also shown. (b) Mean and comparative values of the six parameters included in the PCA for each cluster.

Two uncorrelated factors accounted for 72.55% of co‐variation among parameters measured in the 18 study sites (Figure 5a; Appendix S1: Section S9). Elevated TB seroprevalences, relative abundance of red deer, and network centrality of red deer and wild boar, as well as lower latitudes, were associated with higher factor 1 values. Higher factor 2 values were indicative of increased aggregation of wild boar, increased centrality of red deer, and moderate TB rates (Figure 5a). Four epidemiological clusters were identified. Cluster 1 (*n* = 7) had no TB seroprevalence and low host aggregation and connectivity and was located at higher latitudes. Cluster 2 (*n* = 1) showed the highest seroprevalence, high red deer abundance and shedding‐weighted connectivity, and moderate wild boar aggregation, located in the south. Cluster 3 (*n* = 5) combined low seroprevalence with high wild boar aggregation but low host shedding‐weighted connectivity. Finally, Cluster 4 (*n* = 5) showed high seroprevalence, wild boar aggregation, red deer abundance, and host shedding‐weighted connectivity, as well as low latitude.

## DISCUSSION

This study applies a novel community‐level network analysis combining species co‐occurrence patterns with species‐specific shedding to estimate potential MTC transmission opportunities. By weighing interspecies links with shedding data, we identified high‐risk species and areas in 18 complex multihost systems. Our results suggested that TB risk may depend on species' local network centrality and population factors, whose relative importance varied among species. To our knowledge, this is the first study to integrate species‐level shedding or infectiousness information into ecological co‐occurrence networks, allowing network measures to better capture heterogeneity in transmission potential rather than contact structure alone.

### Mean node centrality measures and their relationship with TB risk

Although our networks represent proxies of ecological interactions and transmission potential, the results suggested that TB risk increased in communities where infectious hosts overlapped more frequently with other susceptible species, as the mean community strength‐out (here a measure of infectious pressure exerted by the host community) was positively related to TB risk. This was consistent with previous studies showing that frequent interspecific connections can facilitate pathogen transmission within ecological communities (Barroso & Gortázar, 2024; Delmas et al., 2019; Su et al., 2022).

### The role of shedding‐weighted centrality and population factors in TB risk

At the species level, models that only included shedding‐weighted centrality values revealed that local connectivity of both wildlife and livestock was associated with TB risk (i.e., strength‐out of wild boar, red deer, and cattle, and closeness of badgers and red foxes). This suggested that multiple species may contribute to TB maintenance in shared environments (Barasona et al., 2014; Ferreira et al., 2024; Nugent et al., 2015; Triguero‐Ocaña et al., 2020). Among them, cattle shedding‐weighted centrality showed the strongest association with TB prevalence, suggesting their importance in transmission dynamics. Although badgers and red foxes are considered minor or context‐dependent hosts in the Iberian Peninsula, our results highlighted the importance of considering even species with lower prevalence that are well connected within the network when designing control measures (Bezos et al., 2023; Tompkins et al., 2011). While our results should be interpreted with caution given the small network sizes, they may have broader relevance in regions with higher badger densities and shedding rates, such as the United Kingdom or Ireland (Holmes et al., 2025; More, 2019).

However, when we included population‐related factors in the models, the importance of species connectivity varied among them. TB risk in the host community was positively associated with population factors such as wild boar aggregation and red deer relative abundance. In this regard, the persistence and spread of the MTC in multispecies ecosystems largely depend on the density of maintenance host species (Gortázar et al., 2012; Renwick et al., 2007). Regarding wild boar, both aggregation and shedding‐weighted connectivity (strength‐out) were significant predictors of TB risk when included simultaneously in the model (see Appendix S1: Section S6, Table S34), suggesting that each variable explained complementary aspects of transmission risk. Specifically, aggregation is related to local density and elevated direct contacts, which likely increase transmission and environmental contamination (Acevedo et al., 2007; Vicente et al., 2013). On the other hand, strength‐out represents the infection pressure exerted by wild boar through their interactions with multiple species in the host community, potentially leading to a broader indirect dissemination of MTC. In contrast, for red deer, the inclusion of both shedding‐weighted connectivity and abundance in the same model revealed that abundance remained the stronger predictor of community‐level TB risk, while centrality lost significance (see Appendix S1: Section S6, Table S35). This suggests that the contribution of red deer to TB risk is mainly driven by the number of individuals present, rather than by their infectious pressure linked to ecological network position, which likely results from population abundance itself instead of independent behavioral or ecological factors (Faisal et al., 2010). This was the opposite of what we observed for badgers and foxes, where network position was more important than population size in explaining TB risk, which could be related to ecological factors such as movement, social organization, or habitat disturbance, as suggested by previous studies (Barroso et al., 2022; Vicente, Delahay, et al., 2007; Wright et al., 2015). A similar result was found in cattle, but in this case spatiotemporal overlap with other species may be related to management‐related factors that facilitate frequent interactions (free‐ranging systems, poor biosecurity, use of shared pastures, etc.), as previously reported (Ferreira et al., 2024; Martínez‐Guijosa et al., 2021). Although our study did not directly assess these drivers, identifying management‐related factors as intervention points (e.g., improving biosecurity measures, controlling pasture use, and limiting opportunities for contact) could help reduce transmission risk (Cowie et al., 2014; Martínez‐Guijosa et al., 2021).

These species‐specific patterns suggested that TB transmission may follow different pathways across host species, and thus, control measures should be adapted accordingly. For red deer, reducing density through targeted culling may be effective, while for wild boar, interventions aimed at reducing aggregation or interspecies contact may be more appropriate (Fattorini et al., 2020; Ueno et al., 2010; Vicente, Delahay, et al., 2007). However, the success of such approaches depends on local population dynamics, compensatory responses, and effort (Fattorini et al., 2020; Prentice et al., 2019).

Finally, although global measures such as betweenness and flow betweenness showed elevated values in some species, they were not associated with community TB risk. Consequently, global centrality measures commonly used in social network analysis may not capture relevant transmission dynamics in this ecological context (Weber et al., 2013).

### Eco‐epidemiological scenarios of TB risk in the Iberian Peninsula

The clustering of host communities suggested four eco‐epidemiological scenarios, also supported by the notable variation in model random intercepts (i.e., study site) even after accounting for population factors and shedding‐weighted connectivity (Appendix S1: Section S7). Clusters 4 and 2 shared high‐risk features (elevated seroprevalence, red deer abundance, and infection pressure of red deer and wild boar), consistent with strong exposure to MTC in dense and connected host communities in southern regions (Barroso et al., 2020; Castillo et al., 2011; Vicente et al., 2013). However, Cluster 4 exhibited increased wild boar aggregation and included multiple sites with consistent TB circulation, while Cluster 2 was a single site with moderate wild boar aggregation but with the highest seroprevalence and infectious pressure. Cluster 3 combined low seroprevalence with high wild boar aggregation but low infection pressure, suggesting that aggregation alone does not necessarily increase TB transmission without high host connectivity or excretion. Cluster 1, dominated by northern sites, represented a low‐risk scenario with no MTC antibodies detected, low host abundance and aggregation, and minimal host connectivity. Although causality cannot be inferred, these findings suggested that geographic and ecological gradients, along with local drivers (e.g., environmental persistence, climate, or management practices), may modulate TB risk (Barroso et al., 2023; Mentaberre et al., 2014; Vicente et al., 2013). For example, in contrast to the north of the Iberian Peninsula, the south‐central regions tend to have higher densities of wild ungulates, which are often managed more intensively within fenced hunting estates, sometimes with supplemental feeding (Vicente, Höfle, et al., 2007). These scenarios, while based on TB, may also reflect broader patterns relevant to other multihost pathogen systems, but further research is needed to validate them across different contexts.

### Limitations

This study has several limitations, including the analysis of only 18 sites, and the need to extrapolate excretion rates for some species. While our conclusions were robust to variation in assigned shedding values, this does not diminish the value of weighting network metrics by species‐specific shedding, which remains important for capturing epidemiological relevance. Furthermore, wild boar TB seroprevalence might not fully capture community infection risk in areas with low density or uneven distribution, or when other species play a larger role in transmission. In such cases, monitoring additional host species alongside wild boar would help improve understanding of infection dynamics. Camera traps identified species but not individuals, so our networks represented spatiotemporal overlap between species as a proxy for potential TB‐relevant contacts. Some studies have successfully used camera traps to identify individuals based on distinctive features (e.g., Egan et al., 2023), but this was not feasible here. However, as camera trapping technology progresses and expands to more species, individual identification may soon become possible. This would offer new opportunities to address current gaps in disease community ecology research. Furthermore, camera trap data are static, meaning that contact detection is limited to the areas where the cameras are placed, which may influence the results. Future studies could address these issues by increasing the number of study sites, using complementary tracking methods, and incorporating more accurate local shedding estimates and individual identification methods. Our observed networks represent only a sample of the underlying co‐occurrence network, and therefore, some sampling error may be present in the calculated network measures. We believe that the sampling strategy and sizeable sample of recorded co‐occurrences support the representativeness of the observed network and the robustness of our results. However, future work could additionally model the observation process and estimating the underlying (latent) co‐occurrence network with uncertainty to quantify how sampling affects the structure of such networks. Finally, we did not perform cross‐validation or out‐of‐sample prediction, as our goal was to explore ecological associations and mechanisms determining TB risk in multihost communities. Additionally, given the relatively small sample sizes at the site level, we prioritized maximizing data use for model fitting. Future studies incorporating additional sites with more diverse host communities and larger datasets could explicitly test and validate the models presented here, enabling out‐of‐sample prediction and improving model robustness.

## CONCLUSIONS

Our results suggest that TB risk in multihost communities may be determined by variation in host abundance, aggregation, and spatiotemporal co‐occurrence of species weighted by their shedding capacity, which we interpret here as a proxy for infection pressure. The importance of these factors varied depending on the species: in the case of wild boar, independent effects of aggregation and connectivity were observed, meaning that centrality was not explained by population‐related factors; as for red deer, connectivity appeared to be driven, at least in part, by abundance; while in the case of badgers, foxes, and cattle, community TB risk was determined solely by their shedding‐weighted connectivity, which could be related to ecological, behavioral, and management factors. Therefore, both wildlife and cattle seem relevant to community‐level TB risk. The species‐specific patterns observed emphasize the need for targeted surveillance and control strategies that consider shedding and interspecies contact structure, which ultimately depend on the context, host ecology, and management.

Compared with previous studies conducted in the same 18 host communities, our approach combines data on population factors and network centrality, considering the opportunities for direct and indirect transmission created by interspecific overlap and shedding (used as an indicator of infection pressure), as well as the resulting structure of multihost communities. It may offer new insights into pathogen maintenance in multispecies systems beyond the Iberian Peninsula and provides a valuable framework for managing TB and other shared diseases in diverse ecological contexts.

## AUTHOR CONTRIBUTIONS

Patricia Barroso contributed to conceptualization, data curation, formal analysis, investigation, methodology, visualization, funding acquisition, writing—original draft. Matthew J. Silk contributed to conceptualization, data curation, investigation, methodology, supervision, validation, visualization, writing—original draft, writing—review and editing. Alberto Perelló contributed to investigation, writing—review and editing. Ana Balseiro contributed to funding acquisition, project administration, resources, supervision, validation, writing—review and editing. Nuno Santos contributed to methodology, writing—review and editing. David Relimpio contributed to investigation, writing—review and editing. Christian Gortázar contributed to conceptualization, funding acquisition, validation, methodology, project administration, resources, supervision, validation, writing—review and editing.

## CONFLICT OF INTEREST STATEMENT

The authors declare no conflicts of interest.

## Supporting information


Appendix S1.

## Data Availability

Camera trap data (Barroso, 2025) are available in Zenodo at https://doi.org/10.5281/zenodo.16568710. Network and population variables and R code (Barroso, 2026) are available in Zenodo at https://doi.org/10.5281/zenodo.18196874.
