# Supplementary material for: Shedding‐weighted network approaches for understanding tuberculosis maintenance in multihost systems using camera traps
Source: Ecol Appl. 2026 Jun 4;36(4):e70274. doi: 10.1002/eap.70274 (PMC13238308; doi:10.1002/eap.70274)
Supplement: Supplementary file 1 — Appendix S1. [file EAP-36-e70274-s001.pdf]

Appendix S1

Shedding-weighted network approaches for understanding tuberculosis maintenance in multihost systems using camera traps

Patricia Barroso, Matthw J. Silk, Alberto Perelló, Ana Balseiro, David Relimpio, Nuno Santos, Christian Gortázar

Ecological Applications

Section S1. Effort (camera-days) by study site

Table S1. Effort (camera-days) by study site (Pto_code). N_camaras means the number of camera traps deployed in the field.

Pto_code	Effort	N_camaras
ALA	450	39
AS	456	18
BAR	394	12
BCN	450	24
BRAG	335	15
CC	450	20
CRE	450	16
DN	450	26
GA	450	16
GRE	450	20
GUA	480	13
HUES	456	15
JLL	421	18
MO	455	13
MUR	450	36
PAL	335	15
PN	450	20
QM	456	19

Section S2. Results of the set of generalized linear mixed models examining the relationship between seropositivity to *Mycobacterium tuberculosis* complex in wild boar as the indicator species of the whole host community and mean local and global centrality measures of species.

Summary of new shedding values

**Species**	**Assigned values MTC concentration (CFU/ml)**
Roe deer	0.90
Red fox	0.90
Egyptian mongoose	0.90
Mouflon	0.90
Domestic small ruminants (sheep and goat)	1.20

Table S2. Results of the generalized linear mixed model examining the relationship between seropositivity to *Mycobacterium tuberculosis* complex in wild boar as the indicator species of the whole host community and mean strength-out of species within the community.

Predictors	***Log-Odds***	***CI***	***p***
(Intercept)	-3.60	-4.99 – -2.21	**<0.001**
Mean strength out	1.39	0.31 – 2.48	**0.012**
Marginal R^2 / Conditional R^2	0.194 / 0.672		
AICc	657.6		

* Statistical significance was set at $p \leq 0.013$ according to Bonferroni correction

Table S3. Results of the generalized linear mixed model examining the relationship between seropositivity to *Mycobacterium tuberculosis* complex in wild boar as the indicator species of the whole host community and mean closeness of species within the community.

Predictors	*Log-Odds*	*CI*	*p*
(Intercept)	-3.62	-5.10 – -2.14	**<0.001**
Mean closeness	1.16	0.05 – 2.28	0.040
Marginal R^2 / Conditional R^2	0.132 / 0.680		
AICc	659.6		

* Statistical significance was set at $p \leq 0.013$ according to Bonferroni correction

Table S4. Results of the generalized linear mixed model examining the relationship between seropositivity to *Mycobacterium tuberculosis* complex in wild boar as the indicator species of the whole host community and mean betweenness of species within the community.

Predictors	***Log-Odds***	***CI***	***p***
(Intercept)	-3.71	-5.41 – -2.01	**<0.001**
Mean betweenness	0.41	-1.14 – 1.96	0.606
Marginal R^2 / Conditional R^2	0.015 / 0.705		
AICc	663.5		

* Statistical significance was set at $p \leq 0.013$ according to Bonferroni correction

Table S5. Results of the generalized linear mixed model examining the relationship between seropositivity to *Mycobacterium tuberculosis* complex in wild boar as the indicator species of the whole host community and mean flow betweenness of species within the community.

Predictors	***Log-Odds***	***CI***	***p***
(Intercept)	-3.69	-5.27 – -2.11	**<0.001**

Mean flow betweenness	0.97	-0.29 – 2.23	0.131
Marginal R^2 / Conditional R^2	0.088 / 0.692		
**AICc**	661.5		

* Statistical significance was set at $p \leq 0.013$ according to Bonferroni correction

Table S6. Results of the generalized linear mixed model examining the relationship between seropositivity to *Mycobacterium tuberculosis* complex in wild boar as the indicator species of the whole host community and closeness of red fox.

Predictors	***Log-Odds***	***CI***	***p***
(Intercept)	-3.63	-5.07 – -2.18	**<0.001**
Closeness of red fox	1.23	0.11 – 2.35	0.015
Marginal R^2 / Conditional R^2	0.250 / 0.651		
cAIC	660.1		

Statistical significance was set at $p \leq 0.013$ according to Bonferroni corrections

Table S7. Results of the generalized linear mixed model examining the relationship between seropositivity to *Mycobacterium tuberculosis* complex in wild boar as the indicator species of the whole host community and flow betweenness of fallow deer.

Predictors	***Log-Odds***	***CI***	***p***
(Intercept)	-2.16	-3.90 – -0.42	**0.015**
Flow betweenness of fallow deer	0.71	-0.72 – 2.14	0.329
**Marginal R^2 / Conditional R^2**	0.072 / 0.530		

Table S8. Results of the generalized linear mixed model examining the relationship between seropositivity to *Mycobacterium tuberculosis* complex in wild boar as the indicator species of the whole host community and closeness of fallow deer.

Predictors	***Log-Odds***	***CI***	***p***
(Intercept)	-2.18	-3.82 – -0.53	**0.009**
Closeness of fallow deer	0.86	-0.48 – 2.19	0.209
**Marginal R^2 / Conditional R^2**	0.108 / 0.515		

Table S9. Results of the generalized linear mixed model examining the relationship between seropositivity to *Mycobacterium tuberculosis* complex in wild boar as the indicator species of the whole host community and strength of fallow deer.

Predictors	***Log-Odds***	***CI***	***p***
(Intercept)	-2.18	-3.83 – -0.53	**0.009**
Strength out of fallow deer	0.85	-0.49 – 2.19	0.213
**Marginal R^2 / Conditional R^2**	0.107 / 0.515		

Table S10. Results of the generalized linear mixed model examining the relationship between seropositivity to *Mycobacterium tuberculosis* complex in wild boar as the indicator species of the whole host community and flow betweenness of roe deer.

Predictors	***Log-Odds***	***CI***	***p***
(Intercept)	-3.52	-5.25 – -1.79	**<0.001**

Flow betweenness of roe deer	-1.60	-3.34 – 0.15	0.073
**Marginal R^2 / Conditional R^2**	0.222 / 0.713		

Table S11. Results of the generalized linear mixed model examining the relationship between seropositivity to *Mycobacterium tuberculosis* complex in wild boar as the indicator species of the whole host community and betweenness of roe deer.

Predictors	***Log-Odds***	***CI***	***p***
(Intercept)	-3.53	-5.13 – -1.94	**<0.001**
Betweenness of roe deer	-1.69	-3.43 – 0.05	0.057
**Marginal R^2 / Conditional R^2**	0.266 / 0.694		

Table S12. Results of the generalized linear mixed model examining the relationship between seropositivity to *Mycobacterium tuberculosis* complex in wild boar as the indicator species of the whole host community and closeness of roe deer.

Predictors	***Log-Odds***	***CI***	***p***
(Intercept)	-3.70	-5.71 – -1.69	**<0.001**
Closeness of roe deer	0.13	-1.64 – 1.91	0.884
**Marginal R^2 / Conditional R^2**	0.002 / 0.702		

Table S13. Results of the generalized linear mixed model examining the relationship between seropositivity to *Mycobacterium tuberculosis* complex in wild boar as the indicator species of the whole host community and strength of roe deer.

Predictors	***Log-Odds***	***CI***	***p***
(Intercept)	-3.49	-5.29 – -1.69	**<0.001**
Strength out of roe deer	-1.55	-3.67 – 0.58	0.155
**Marginal R^2 / Conditional R^2**	0.204 / 0.720		

Table S14. Results of the generalized linear mixed model examining the relationship between seropositivity to *Mycobacterium tuberculosis* complex in wild boar as the indicator species of the whole host community and flow betweenness of small ruminants.

Predictors	***Log-Odds***	***CI***	***p***
(Intercept)	-5.00	-6.36 – -3.64	**<0.001**
Flow betweenness of small ruminants	-0.61	-2.67 – 1.44	0.558
**Marginal R^2 / Conditional R^2**	0.103 / NA		

Table S15. Results of the generalized linear mixed model examining the relationship between seropositivity to *Mycobacterium tuberculosis* complex in wild boar as the indicator species of the whole host community and betweenness of small ruminants.

Predictors	***Log-Odds***	***CI***	***p***
(Intercept)	-5.34	-6.95 – -3.73	**<0.001**
Betweenness of small ruminants	0.85	-0.10 – 1.80	0.080
**Marginal R^2 / Conditional R^2**	0.180 / NA		

Table S16. Results of the generalized linear mixed model examining the relationship between seropositivity to *Mycobacterium tuberculosis* complex in wild boar as the indicator species of the whole host community and closeness of small ruminants.

Predictors	***Log-Odds***	***CI***	***p***
(Intercept)	-7.19	-12.22 – -2.16	**0.005**
Closeness of small ruminants	-4.82	-13.30 – 3.66	0.265
**Marginal R^2 / Conditional R^2**	0.876 / NA		

Table S17. Results of the generalized linear mixed model examining the relationship between seropositivity to *Mycobacterium tuberculosis* complex in wild boar as the indicator species of the whole host community and strength of small ruminants.

Predictors	***Log-Odds***	***CI***	***p***
(Intercept)	-4.93	-6.15 – -3.70	**<0.001**
Strength out of small ruminants	-0.39	-1.98 – 1.20	0.633
**Marginal R^2 / Conditional R^2**	0.044 / NA		

Section S3. Node centrality measures by species and study site.

Table S18. Node centrality measures (degree, strength out, closeness, betweenness, flow betweenness) by species and study site.

Species	Degree	StrengthOut	WeightCloseness	WeightBetweenness	Flow_bet	Study site
Cattle	8	36.01	0.1110109	10	46.11	ALA
Roedeer	10	49.32	0.1058118	16	226.73	ALA
Reddeer	2	2.038	0.2462162	0	0	ALA
Fallowdeer	2	2.988	0.0938514	0	0	ALA
Europeanbadger	8	155.595	0.1075738	8	145.635	ALA
Wildboar	6	979.352	0.1080977	18	271.215	ALA
Redfox	8	31.92	0.1027343	0	93.915	ALA
Cattle	10	78.945	2.5730813	8	154.833	AS
Roedeer	10	19.2	1.068786	0	95.36	AS
Reddeer	10	240.484	3.4570798	12	265.206	AS
Europeanbadger	10	16.445	0.9664183	0	44.405	AS
Wildboar	10	301.56	3.135314	4	165.524	AS
Redfox	10	6.84	0.5164486	0	44.8	AS
Cattle	8	40.165	1.1883503	0	95.212	BAR
Reddeer	12	175.268	2.2646503	15	323.569	BAR
Egyptianmongoose	8	2.04	0.1140061	0	14.122	BAR
Europeanbadger	10	80.96	1.6839802	4	177.682	BAR
SmallRuminant	4	3.24	0.2556173	0	6.48	BAR
Wildboar	12	212.528	2.3894397	12	208.696	BAR
Redfox	10	12.96	0.5308301	0	88.882	BAR
Roedeer	10	14.76	1.1464758	0	89.753	BRAG
Reddeer	10	232.332	4.0264202	9	231.299	BRAG
Europeanbadger	10	83.49	1.8400104	3	121.81	BRAG
SmallRuminant	10	42.93	1.0265669	0	87.213	BRAG
Wildboar	10	143.6	3.6686131	0	127.631	BRAG
Redfox	10	17.04	0.7892898	1	131.076	BRAG
Roedeer	2	1.08	0.1737611	0	0	CC
Reddeer	12	970.088	3.9371377	29	819.677	CC
Egyptianmongoose	6	1.68	0.1552155	0	7.635	CC
Europeanbadger	8	78.43	2.8865425	0	55.227	CC
Mouflon	6	1.08	0.0791837	0	3	CC
Wildboar	8	697.896	3.8220491	0	125.652	CC
Redfox	10	29.4	1.9938659	0	63.1	CC
Reddeer	4	18.342	2.1237794	0	2.158	CRE
Wildboar	4	25.848	2.5528889	1	2.992	CRE
Redfox	4	0.24	0.06	0	0.24	CRE
Cattle	4	126.035	1.2010641	0	46.827	DN

Reddeer	12	295.51	1.2745724	26	379.429	DN
Fallowdeer	6	16.434	0.7650907	0	23.719	DN
Egyptianmongoose	2	0.12	0.0198831	0	0	DN
Europeanbadger	6	7.59	0.5412197	0	12.004	DN
Wildboar	10	215.4	1.2770538	14	162.931	DN
Redfox	8	1.32	0.0935621	0	11.28	DN
Cattle	6	5.54	0.0900588	0	9.385	GA
Roedeer	8	1.2	0.05803	8	8.045	GA
Europeanbadger	2	1.265	0.0778107	0	0	GA
SmallRuminant	6	9.72	0.1020271	4	12.99	GA
Wildboar	8	43.08	0.1102143	15	29.471	GA
Redfox	6	0.72	0.0373405	0	5.035	GA
Reddeer	10	179.344	4.5252054	20	234.703	GRE
Roedeer	10	6.84	0.7155936	0	41.868	GRE
Wildboar	6	40.208	2.8900779	0	13.408	GRE
Europeanbadger	4	8.855	1.0948551	0	3.97	GRE
Cattle	6	23.545	2.0792742	0	10.285	GRE
Redfox	8	5.16	0.554116	0	20.892	GRE
Reddeer	12	167.116	2.0966433	21	242.231	GUA
Roedeer	12	12.36	0.5618515	5	71.132	GUA
Fallowdeer	10	44.82	1.1460876	0	77.316	GUA
Wildboar	10	186.68	2.1381532	6	153.622	GUA
SmallRuminant	4	4.05	0.4532813	0	1.77	GUA
Europeanbadger	10	10.12	0.3826174	0	29.156	GUA
Redfox	10	4.44	0.2420853	0	30.774	GUA
Roedeer	4	4.68	1.1262745	0	1.08	HUES
Wildboar	6	160.832	4.9234286	6	18.74	HUES
Europeanbadger	2	2.53	0.8231899	0	0	HUES
Redfox	4	2.52	0.6358672	0	1.08	HUES
Reddeer	6	1760.832	20.90702	2	91.156	JLL
Wildboar	6	2492.896	21.518387	5	150.492	JLL
Europeanbadger	4	16.445	4.019178	0	9.909	JLL
Redfox	4	7.8	1.2965129	0	7.8	JLL
Reddeer	8	1520.348	3.9438654	12	760.168	MO
Wildboar	8	1539.392	3.9106604	0	373.403	MO
Mouflon	6	35.28	2.4988921	0	39.6	MO
Europeanbadger	4	3.795	0.6292286	0	3.795	MO
Redfox	6	20.52	1.727912	0	24.84	MO
Europeanbadger	4	6.325	1.6760326	0	2.77	MUR
Wildboar	4	22.976	5.385	2	4.395	MUR
Redfox	4	0.84	0.2899058	0	0.84	MUR
Cattle	4	44.32	0.4256236	0	11.52	PAL
Roedeer	12	40.68	0.4089014	17	227.79	PAL
Reddeer	4	12.228	0.7311419	0	2.28	PAL
Europeanbadger	4	116.38	0.4354573	0	29.4	PAL

SmallRuminant	6	23.49	0.3600477	0	37.11	PAL
Wildboar	6	493.984	0.4576396	5	134.196	PAL
Redfox	12	40.32	0.4012207	17	248.93	PAL
Reddeer	10	4073.962	22.980504	20	4305.366	PN
Roedeer	6	4.8	0.7744299	0	8.4	PN
Fallowdeer	6	1640.412	21.559781	0	467.424	PN
Wildboar	10	2805.944	22.233363	0	613.02	PN
Europeanbadger	8	45.54	4.9782481	0	66.462	PN
Redfox	8	13.92	2.0564803	0	22.94	PN
Reddeer	8	474.854	3.0776394	12	225.534	QM
Roedeer	2	0.84	0.1993329	0	0	QM
Fallowdeer	6	5.976	0.7050824	0	7.714	QM
Wildboar	6	422.184	3.0190264	0	19.562	QM
Redfox	6	10.2	1.369133	0	13.698	QM
Roedeer	4	0.48	0.115128	0	0.24	BCN
Wildboar	6	235.504	2.1334857	5	15.328	BCN
SmallRuminant	4	2.43	0.4583812	0	1.86	BCN
Redfox	6	9.72	1.2742662	2	3.84	BCN

Section S4. Results of the set of generalized linear mixed models examining the relationship between seropositivity to *Mycobacterium tuberculosis* complex in wild boar as the indicator species of the whole host community and mean local and global centrality measures of species.

Table S19. Results of the generalized linear mixed model examining the relationship between seropositivity to *Mycobacterium tuberculosis* complex in wild boar as the indicator species of the whole host community and mean strength-out of species within the community. R call: glmer(TB ~ Mean_strengthOut+(1|Pto_code), family=binomial (link = "logit"), data=df).

Predictors	***Log-Odds***	***CI***	***p***
(Intercept)	-3.59	-4.97 – -2.20	**<0.001**
Mean strength out	1.40	0.33 – 2.48	**0.010**
Marginal R^2 / Conditional R^2	0.197 / 0.671		
AICc	657.4		

* Statistical significance was set at *p* ≤ 0.013 according to Bonferroni correction

Table S20. Results of the generalized linear mixed model examining the relationship between seropositivity to *Mycobacterium tuberculosis* complex in wild boar as the indicator species of the whole host community and mean closeness of species within the community. R call: glmer(TB ~ Mean_WeightCloseness+(1|Pto_code), family=binomial (link = "logit"), data=df).

Predictors	*Log-Odds*	*CI*	*p*
(Intercept)	-3.62	-5.10 – -2.14	**<0.001**
Mean closeness	1.16	0.05 – 2.28	0.040
Marginal R^2 / Conditional R^2	0.132 / 0.680		
AICc	659.2		

* Statistical significance was set at *p* ≤ 0.013 according to Bonferroni correction

Table S21. Results of the generalized linear mixed model examining the relationship between seropositivity to *Mycobacterium tuberculosis* complex in wild boar as the indicator species of the whole host community and mean betweenness of species within the community. R call: glmer(TB ~ Mean_WeightBetweenness+(1|Pto_code), family=binomial (link = "logit"), data=df).

Predictors	***Log-Odds***	***CI***	***p***
(Intercept)	-3.73	-5.44 – -2.01	**<0.001**
Mean betweenness	0.44	-1.06 – 1.94	0.563
Marginal R^2 / Conditional R^2	0.017 / 0.707		
AICc	663.4		

* Statistical significance was set at *p* ≤ 0.013 according to Bonferroni correction

Table S22. Results of the generalized linear mixed model examining the relationship between seropositivity to *Mycobacterium tuberculosis* complex in wild boar as the indicator species of the whole host community and mean flow betweenness of species within the community. R call: glmer(TB ~ Mean_Flowbet+(1|Pto_code), family=binomial (link = "logit"), data=df).

Predictors	***Log-Odds***	***CI***	***p***
(Intercept)	-3.64	-5.17 – -2.10	**<0.001**
Mean flow betweenness	1.09	-0.09 – 2.28	0.071
Marginal R^2 / Conditional R^2	0.113 / 0.688		
**AICc**	660.5		

* Statistical significance was set at *p* ≤ 0.013 according to Bonferroni correction

Section S5. Results of model selection processes. Best models are highlighted in bold font. K means number of parameters, including both the intercept and the error terms; AIC Akaike's Information Criterion; Δi difference of AIC with respect to the best model; wi Akaike weight.

Table S23. Models on mean centrality measures

	n	K	AIC	AICc	Δi	L(gi/x)	Wi
**TB ~ strength out**	1109	3	657.4	657.42	0.00	1	0.63
TB ~ closeness	1109	3	659.60	659.62	2.20	0.33	0.21
TB ~ flow betweenness	1109	3	660.50	660.52	3.10	0.21	0.13
TB~ betweenness	1109	3	663.40	663.42	6.00	0.05	0.03

Table S24. Models on wild boar centrality measures

	n	K	AIC	AICc	Δi	L(gi/x)	Wi
**TB ~ strength out of wild boar**	1109	3	658	658.02	0.00	1	0.63
TB ~ closeness of wild boar	1109	3	660.10	660.12	2.10	0.35	0.22
TB ~ flow betweenness of wild boar	1109	3	661.10	661.12	3.10	0.21	0.13
TB ~ betweenness of wild boar	1109	3	662.20	662.22	4.20	0.12	0.08

Table S25. Models on red deer centrality measures

	n	K	AIC	AICc	Δi	L(gi/x)	Wi
**TB ~ strength out of red deer**	1109	3	629.2	629.22	0.00	1	0.63
TB ~ betweenness of red deer	1109	3	630.20	630.22	1.00	0.61	0.38
TB ~ closeness of red deer	1109	3	630.90	630.92	1.70	0.43	0.27
TB ~ flow betweenness of red deer	1109	3	631.90	631.92	2.70	0.26	0.16

Table S26. Models on red fox centrality measures

	n	K	AIC	AICc	Δi	L(gi/x)	Wi
**TB ~ closeness of red fox**	1109	3	655.7	655.72	0	1	0.63
TB ~ betweenness of red fox	1109	3	657	657.02	1.3	0.52	0.33
TB ~ flow betweenness of red fox	1109	3	661.5	661.52	5.8	0.06	0.03
TB ~ strength out of red fox	1109	3	663.7	663.72	8	0.02	0.01

Table S27. Models on European badger centrality measures

	n	K	AIC	AICc	Δi	L(gi/x)	Wi
**TB ~ closeness of European badger**	1109	3	525.8	525.82	0	1	0.63
TB ~ betweenness of European badger	1109	3	527.4	527.42	1.6	0.45	0.28
TB ~ flow betweenness of European badger	1109	3	530.3	530.32	4.5	0.11	0.07
TB ~ strength out of European badger	1109	3	530.7	530.72	4.9	0.09	0.05

Table S28. Models on cattle centrality measures

	n	K	AIC	AICc	Δi	L(gi/x)	Wi
**TB ~ closeness of cattle**	386	3	247.5	247.56	0	1.00	0.63
TB ~ betweenness of cattle	386	3	250.2	250.26	2.7	0.26	0.16
TB ~ flow betweenness of cattle	386	3	252.8	252.86	5.3	0.07	0.04
TB ~ strength out of cattle	386	3	252.8	252.86	5.3	0.07	0.04

Table S29. Model selection on wild boar including population-related factors and centrality measures

Intercept	Aggregation of wild boar	Strength out of wild boar	df	logLik	AICc	Δi	Wi
**-3.46**	**1.34**	**1.14**	4	-323.06	654.16	0	0.70
-3.50	1.76		3	-325.43	656.89	2.73	0.18
-3.62		1.35	3	-326.01	658.037	3.88	0.10
-3.68			2	-328.86	661.73	7.57	0.02

Table S30. Model selection on red deer including population-related factors and centrality measures

Intercept	Relative abundance of red deer	Strength out of red deer	df	logLik	AICc	Δi	Wi
**-2.46**	**0.94**	**0.81**	4	-309.39	626.83	0	0.53
-2.51	1.28		3	-311.04	628.11	1.28	0.28
-2.58		1.25	3	-311.59	629.20	2.37	0.16
-2.66			2	-314.35	632.72	5.89	0.03

Table S31. Model selection on red fox including population-related factors and centrality measures

Intercept	Relative abundance of red fox	Closeness of red fox	df	logLik	AICc	Δi	Wi
**-3.51**		**1.54**	3	-324.85	655.73	0	0.54
-3.50	-1.22	1.62	4	-324.09	656.22	0.49	0.42
-3.68			2	-328.86	661.73	6.00	0.03
-3.57	-0.71		3	-328.29	662.60	6.87	0.02

Table S32. Model selection on European badger including population-related factors and centrality measures

Intercept	Relative abundance of European badger	Closeness of European badger	df	logLik	AICc	Δi	Wi
**-3.41**	**-2.12**	**1.21**	4	-258.17	524.38	0	0.66
-3.28	-2.10		3	-260.58	527.19	2.82	0.16
-3.33		1.22	3	-260.72	527.47	3.08	0.14
-3.39			2	-263.099	530.21	5.83	0.04

Table S33. Model selection on cattle including population-related factors and centrality measures

Intercept	Relative abundance of cattle	Strength-out of cattle	df	logLik	AICc	Δi	Wi
**-2.41**		**0.95**	3	-120.73	247.52	0	0.52
-2.39	-0.22	0.89	4	-120.08	248.26	0.74	0.36
-3.11			2	-123.46	250.96	3.44	0.09
-3.10	-0.16		3	-123.43	252.91	5.40	0.04

Section S6. Results of the set of generalized linear mixed models examining the relationship between seropositivity to *Mycobacterium tuberculosis* complex in wild boar as the indicator species of the whole host community and local and global centrality measures of species.

Table S34. Results of the generalized linear mixed model examining the relationship between seropositivity to *Mycobacterium tuberculosis* complex in wild boar as the indicator species of the whole host community and strength-out of wild boar. R call: glmer(TB ~ StrengthOut_Wildboar+(1|Pto_code), family=binomial (link = "logit"), data=df).

Predictors	*Log-Odds*	*CI*	*p*
(Intercept)	-3.60	-5.03 – -2.22	**<0.001**
Strength out of wild boar	1.39	0.27 – 2.43	**0.010**
Marginal R^2 / Conditional R^2	0.184 / 0.670		
cAIC	658		

Statistical significance was set at $p \leq 0.013$ according to Bonferroni corrections

Table S35. Results of the generalized linear mixed model examining the relationship between seropositivity to *Mycobacterium tuberculosis* complex in wild boar as the indicator species of the whole host community and strength-out of red deer. R call: glmer(TB ~ StrengthOut_Reddeer+(1|Pto_code), family=binomial (link = "logit"), data=df).

Predictors	***Log-Odds***	***CI***	***p***
(Intercept)	-2.58	-3.74 – -1.42	**<0.001**
Strength out of red deer	1.25	0.23 – 2.27	**0.011**
Marginal R^2 / Conditional R^2	0.196 / 0.585		
cAIC	629.2		

Statistical significance was set at $p \leq 0.013$ according to Bonferroni corrections

Table S36. Results of the generalized linear mixed model examining the relationship between seropositivity to *Mycobacterium tuberculosis* complex in wild boar as the indicator species of the whole host community and closeness of red fox. R call: glmer(TB ~ WeightCloseness_Redfox+(1|Pto_code), family=binomial (link = "logit"), data=df).

Predictors	***Log-Odds***	***CI***	***p***
(Intercept)	-3.51	-4.75 – -2.26	**<0.001**
Closeness of red fox	1.54	0.54 – 2.53	**0.002**
Marginal R^2 / Conditional R^2	0.250 / 0.651		
cAIC	660.1		

Statistical significance was set at $p \leq 0.013$ according to Bonferroni corrections

Table S37. Results of the generalized linear mixed model examining the relationship between seropositivity to *Mycobacterium tuberculosis* complex in wild boar as the indicator species of the whole host community and closeness of European badger. R call: glmer(TB ~ WeightCloseness_Europeanbadger+(1|Pto_code), family=binomial (link = "logit"), data=df).

Predictors	***Log-Odds***	***CI***	***p***
(Intercept)	-3.33	-4.61 – -2.04	**<0.001**
Closeness of European badger	1.22	0.21 – 2.23	**0.010**
Marginal R^2 / Conditional R^2	0.175 / 0.614		
cAIC	655.7		

Statistical significance was set at $p \leq 0.013$ according to Bonferroni corrections

Table S38. Results of the generalized linear mixed model examining the relationship between seropositivity to *Mycobacterium tuberculosis* complex in wild boar as the indicator species of

the whole host community and strength-out of cattle. R call: glmer(TB ~ StrengthOut_Cattle+(1|Pto_code), family=binomial (link = "logit"), data=df).

Predictors	***Log-Odds***	***CI***	***p***
(Intercept)	-2.41	-2.83 – -1.99	**<0.001**
Strength out of cattle	0.95	0.56 – 1.34	**<0.001**
**Marginal R^2 / Conditional R^2**	0.214 / 0.214		
cAIC	247.5		

Statistical significance was set at $p \leq 0.013$ according to Bonferroni corrections

Section S7. Random intercepts (conditional modes or Best Linear Unbiased Predictors - BLUPs) for all models included in the manuscript. Site-level random intercepts were extracted using the *lme4* package in R. K-means cluster information from Figure 5a is shown.

TB ~ mean strength out

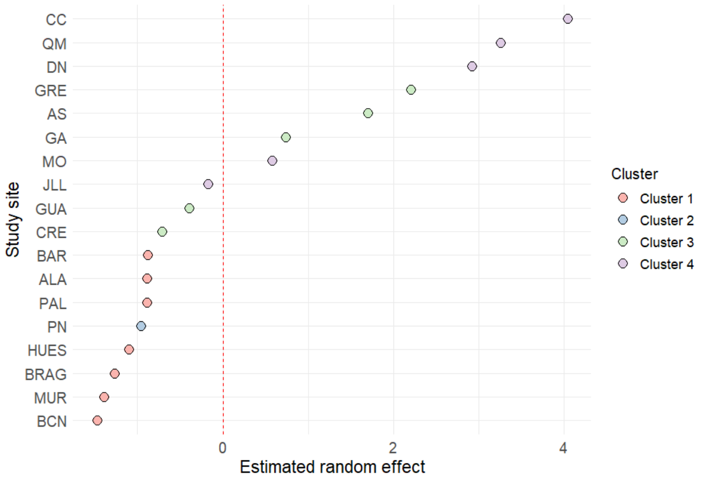

TB ~ mean closeness

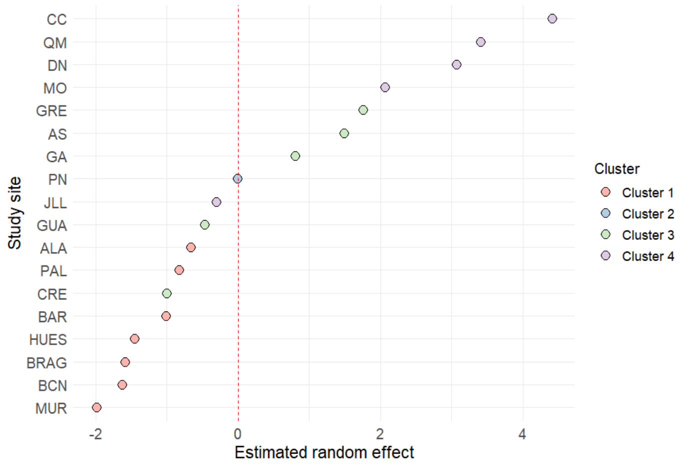

TB ~ strength out of wild boar

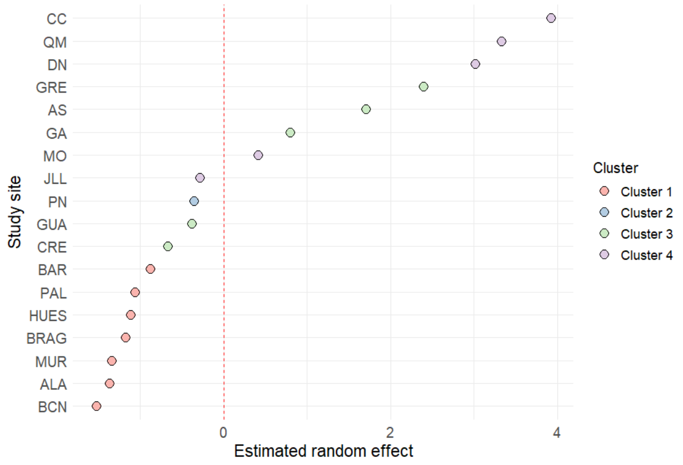

TB ~ closeness of wild boar

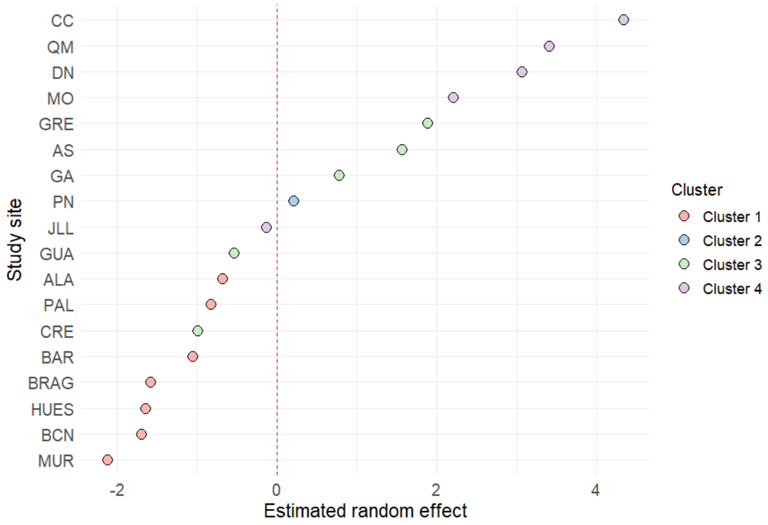

TB ~ strength out of red deer

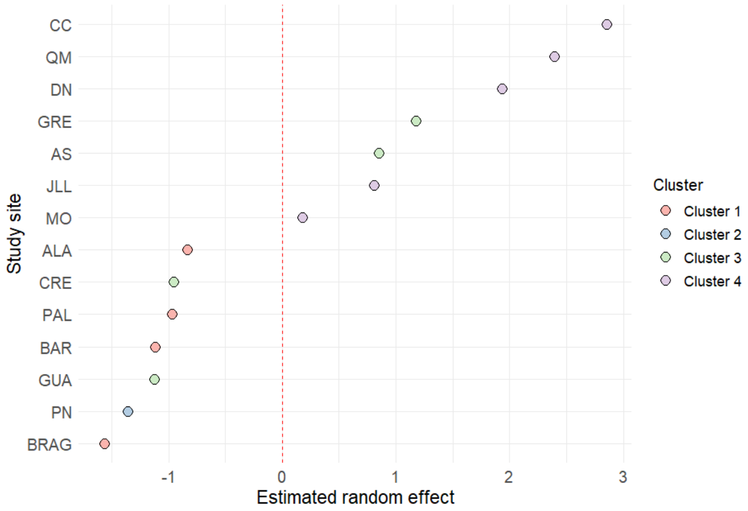

TB ~ betweenness of red deer

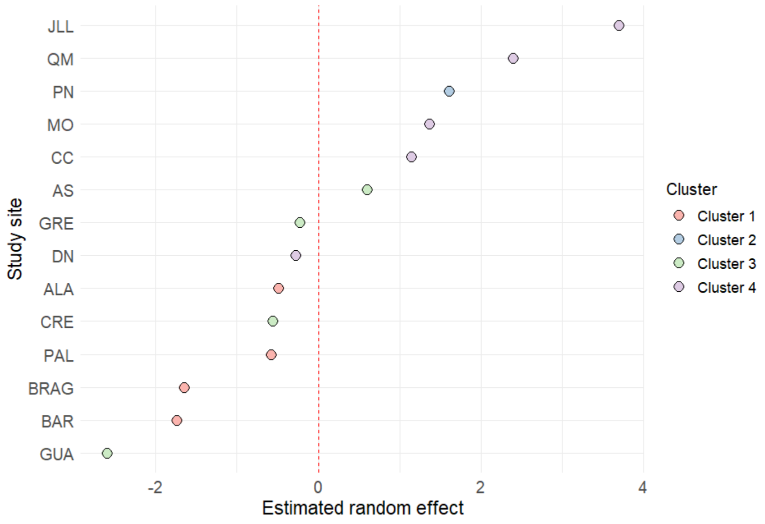

TB ~ closeness of red deer

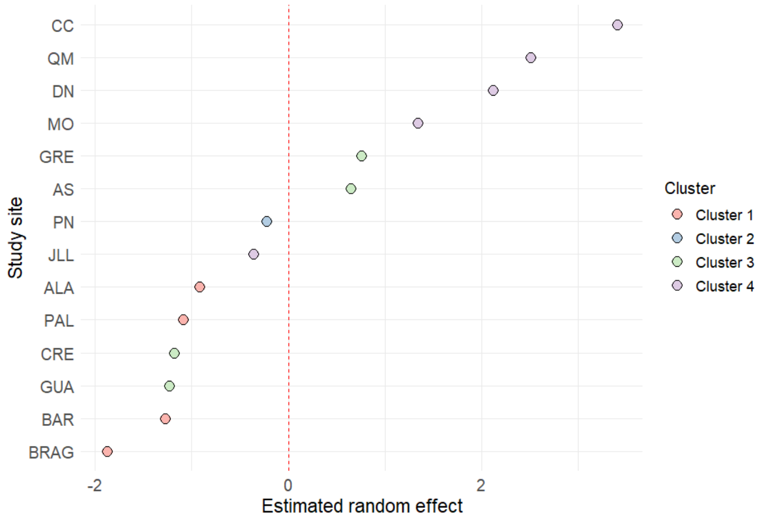

TB ~ strength out of cattle

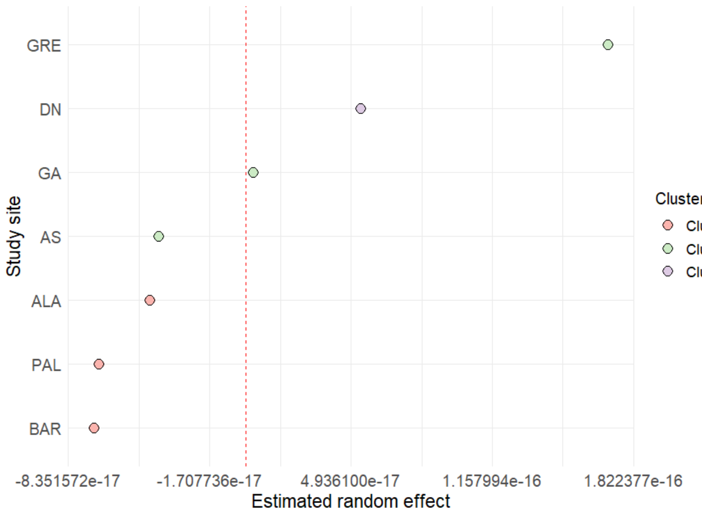

TB ~ closeness of red fox

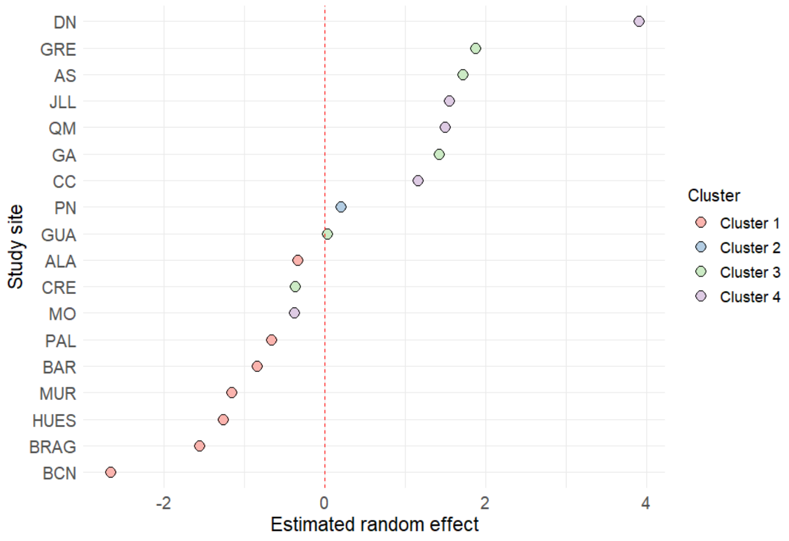

TB ~ closeness of European badger

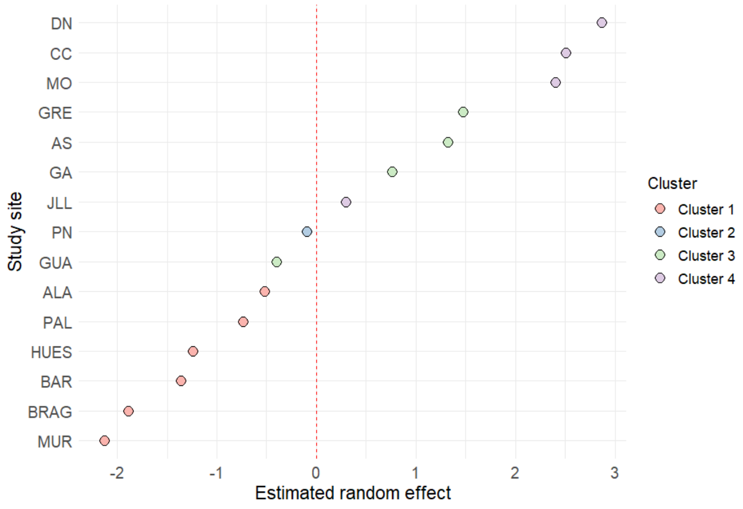

TB ~ strength out of wild boar + aggregation of wild boar

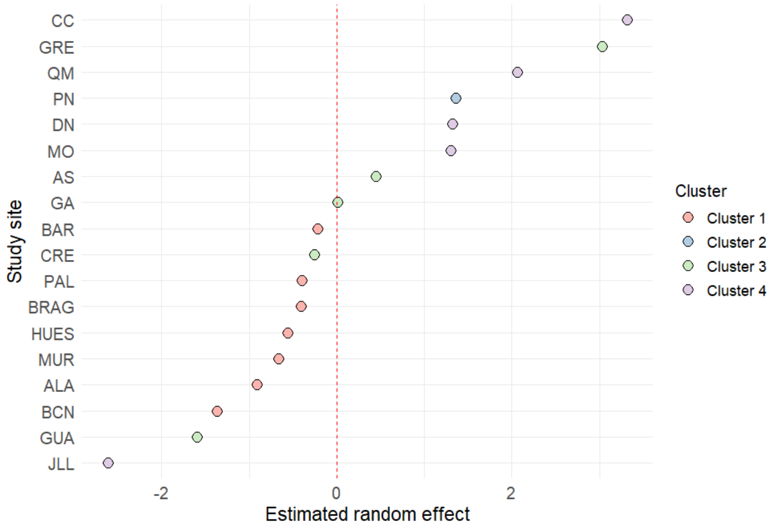

TB ~ strength out of red deer + relative abundance of red deer

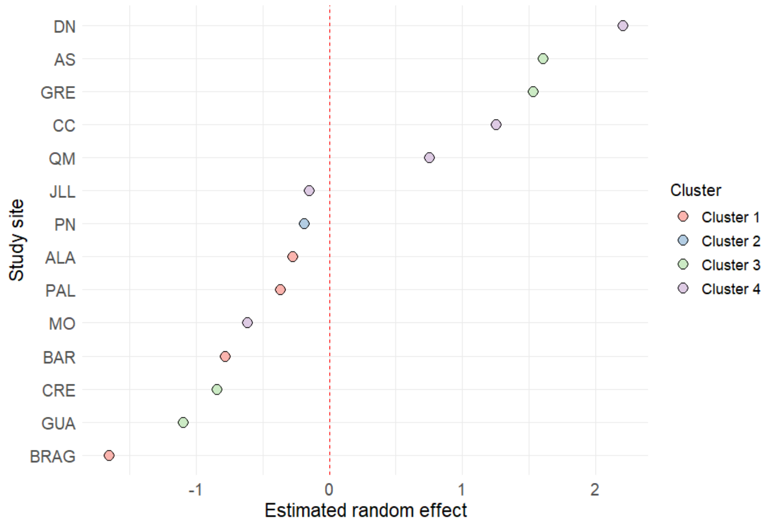

TB ~ closeness of badger + relative abundance of badger

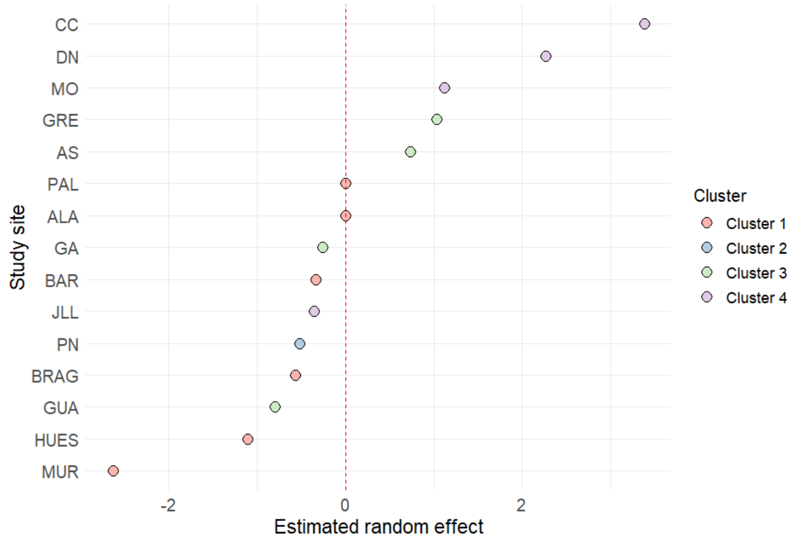

Section S8. Results of the best generalized linear mixed models examining the relationship between seropositivity to *Mycobacterium tuberculosis* complex in wild boar as the indicator species of the whole host community, local centrality measures of species and population-related factors of each species.

Table S39. Results of the best generalized linear mixed model examining the relationship between seropositivity to *Mycobacterium tuberculosis* complex in wild boar as the indicator species of the whole host community, strength-out of wild boar and its aggregation index. R call: glmer(TB ~ StrengthOut_Wildboar+ Agreg.Wildboar+(1|Pto_code), family=binomial(link = "logit"), data=df).

Predictors	***Log-Odds***	***CI***	***p***
(Intercept)	-3.46	-4.74 – -2.18	**<0.001**
Strength out of wild boar	1.14	0.17 – 2.11	**0.021**
Aggregation of wild boar	1.34	0.15 – 2.52	**0.027**
Marginal R^2 / Conditional R^2	0.316 / 0.687		
cAIC	654.2		

Statistical significance was set at $p \leq 0.050$

Table S40. Results of the best generalized linear mixed model examining the relationship between seropositivity to *Mycobacterium tuberculosis* complex in wild boar as the indicator species of the whole host community, strength-out of red deer and its relative abundance (FBII - Frequency-Based Indirect Index). R call: glmer(TB ~ StrengthOut_Reddeer+FBII.reddeer+(1|Pto_code), family=binomial(link = "logit"), data=df).

Predictors	***Log-Odds***	***CI***	***p***
(Intercept)	-2.46	-3.41 – -1.50	**<0.001**
Strength out of red deer	0.81	-0.04 – 1.65	0.061
Relative abundance of red deer (FBII)	0.94	0.09 – 1.78	**0.029**
**Marginal R^2 / Conditional R^2**	0.282 / 0.545		
cAIC	626.9		

Statistical significance was set at $p \leq 0.050$

Table S41. Results of the best generalized linear mixed model examining the relationship between seropositivity to *Mycobacterium tuberculosis* complex in wild boar as the indicator species of the whole host community and closeness of red fox. R call: glmer(TB ~ WeightCloseness_Redfox +(1|Pto_code), family=binomial(link = "logit"), data=df).

Predictors	***Log-Odds***	***CI***	***p***
(Intercept)	-3.51	-4.75 – -2.26	**<0.001**
Closeness of red fox	1.54	0.54 – 2.53	**0.002**
Marginal R^2 / Conditional R^2	0.250 / 0.651		
cAIC	655.7		

Statistical significance was set at $p \leq 0.050$

Table S42. Results of the best generalized linear mixed model examining the relationship between seropositivity to *Mycobacterium tuberculosis* complex in wild boar as the indicator species of the whole host community, closeness of European badger and its relative abundance (trapping rate). R call: glmer(TB ~ WeightCloseness_Europeanbadger+Abund_Europeanbadger+(1|Pto_code), family=binomial(link = "logit"), data=df).

Predictors	***Log-Odds***	***CI***	***p***
(Intercept)	-3.41	-4.78 – -2.04	**<0.001**
Closeness of European badger	1.21	0.23 – 2.20	**0.016**
Relative abundance of European badger	-2.12	-4.74 – 0.50	0.112
Marginal R^2 / Conditional R^2	0.493 / 0.742		
cAIC	524.4		

Statistical significance was set at $p \leq 0.050$

Table S43. Results of the best generalized linear mixed model examining the relationship between seropositivity to *Mycobacterium tuberculosis* complex in wild boar as the indicator species of the whole host community and strength-out of cattle. R call: glmer(TB ~ StrengthOut_Cattle+(1|Pto_code), family=binomial(link = "logit"), data=df).

Predictors	***Log-Odds***	***CI***	***p***
(Intercept)	-2.41	-2.83 – -1.99	**<0.001**
Strength out of cattle	0.95	0.56 – 1.34	**<0.001**
**Marginal R^2 / Conditional R^2**	0.214 / 0.214		
cAIC	247.5		

Statistical significance was set at $p \leq 0.050$

Section S9. Result of the Principal Component Analysis (PCA) and the factors obtained.

Table S44. Result of the Principal Component Analysis (PCA) and the two factors obtained.

Variable	Factor 1	Factor 2
**Tuberculosis seroprevalence**	0.88	0.17
**Strength out of red deer**	0.76	-0.57
**Strength out of wild boar**	0.81	-0.41
**Latitude**	-0.64	-0.08
**Aggregation of wild boar**	0.42	0.69
**Relative abundance of red deer (FBII)**	0.79	0.33
**Eigenvalue**	3.23	1.12
**Explained variance**	53.91	18.64
**Cumulative variance**	53.91	72.55
